# Supplementary material for: pGlyco 2.0 enables precision N-glycoproteomics with comprehensive quality control and one-step mass spectrometry for intact glycopeptide identification
Source: Nat Commun. 2017 Sep 5;8:438. doi: 10.1038/s41467-017-00535-2 (PMC5585273; doi:10.1038/s41467-017-00535-2)
Supplement: Supplementary file 1 — Supplementary Information [file 41467_2017_535_MOESM1_ESM.pdf]

File Name: Supplementary Information
Descriptions: Supplementary Figures, Supplementary Tables, Supplementary Notes and Supplementary References

File Name: Supplementary Data 1
Descriptions: identified glycopeptide spectra in standard glycoprotein mixture.

File Name: Supplementary Data 2
Descriptions: identified glycopeptide spectra in mouse tissues.

File Name: Supplementary Data 3
Descriptions: site-specific glycan information in mouse tissues from glycopeptide data.

File Name: Supplementary Data 4
Descriptions: glycosylation site information in mouse tissues from glycopeptide data.

File Name: Supplementary Data 5
Descriptions: glycoprotein information in mouse tissues from glycopeptide data.

File Name: Peer Review File

Supplementary Figure 1

Comparing spectra of the same glycopeptide from different instruments

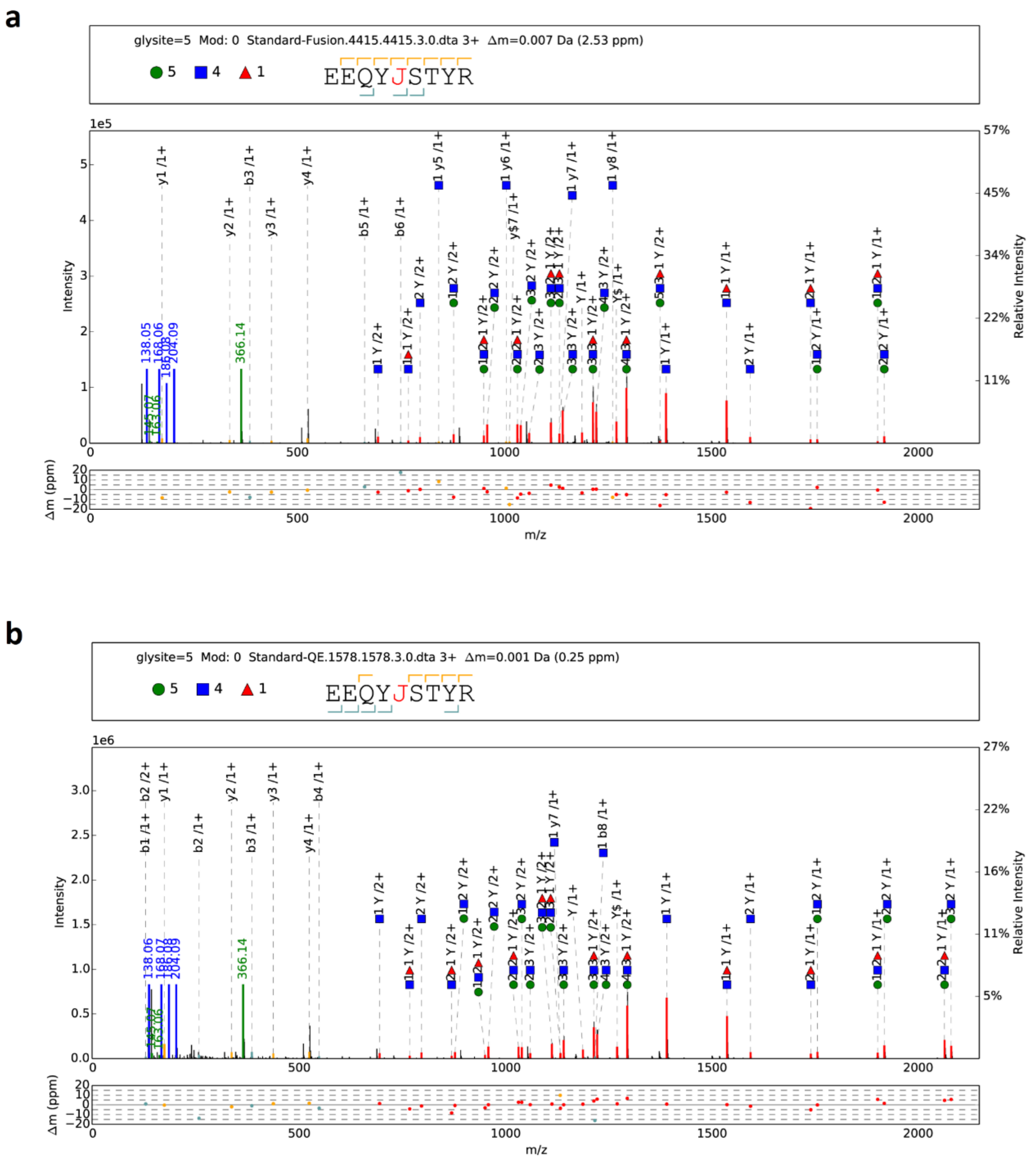

Supplementary Figure 1. Comparing spectra of the same glycopeptide from different instruments. (a) A high-quality N-glycopeptide spectrum derived from SCE-HCD-MS/MS on an Orbitrap Fusion instrument. (b) SCE-HCD-MS/MS spectrum of the same glycopeptide obtained on a Q Exactive

instrument. The design of the upper box above each spectrum is as follows: glycosylation site (glysite); modification (mod); spectral name; precursor mass deviation; glycan composition; and peptide sequence, with “J” indicating the N-glycosylation site. The glycan symbols are green circle for Hex, blue square for HexNAc, purple diamond for NeuAc and red triangle for fucose. Peak annotation is shown in the middle box–green, blue and purple peaks represent the fragment ions of the glycan moiety or the diagnostic glycan ions; red peaks represent the Y ions from glycan fragmentation; and yellow/cyan peaks represent the b/y ions from peptide backbone fragmentation. For clear illustration, the scale of the relative intensity is automatically adjusted based on the highest peak between 700~2,000 Th. Mass deviations of the annotated peaks are shown in the lower box.

Supplementary Figure 2

Illustration of different optimum collision energies for different glycopeptides

a1

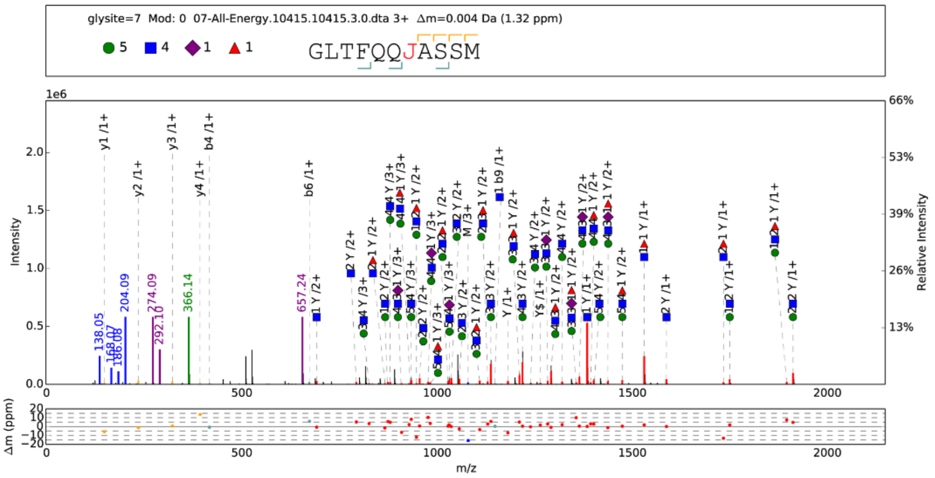

b1

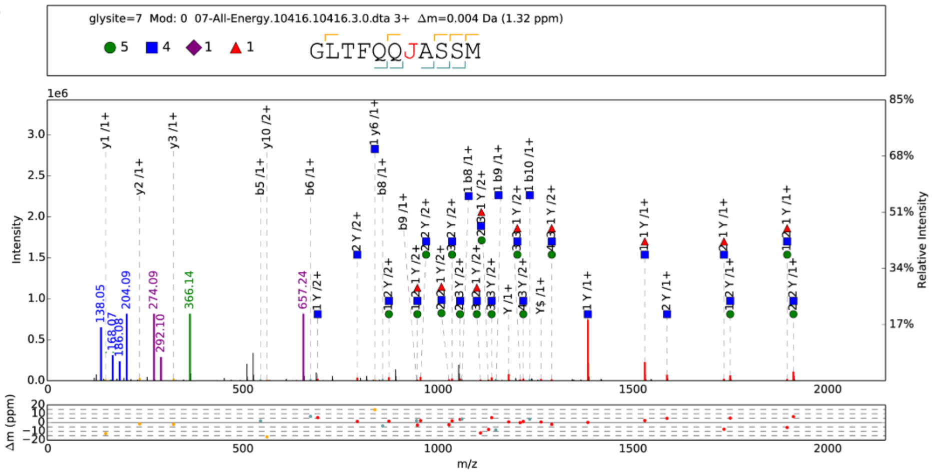

c1

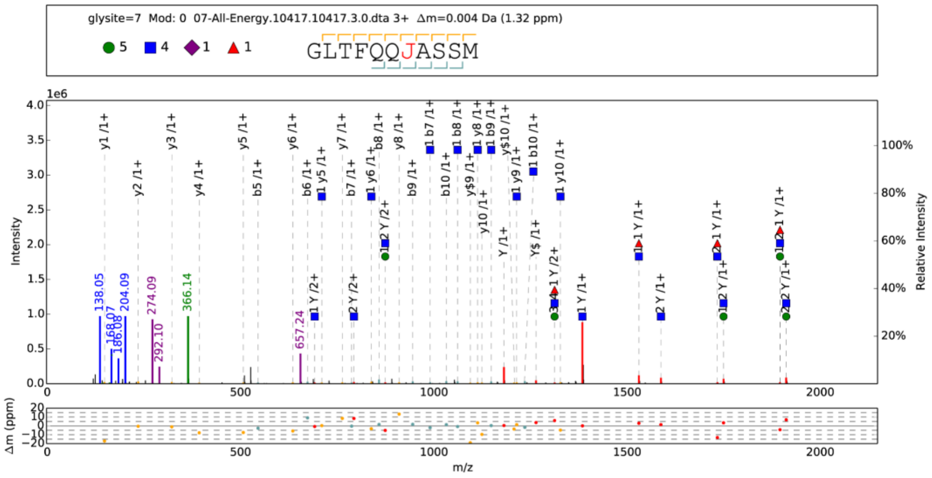

a2

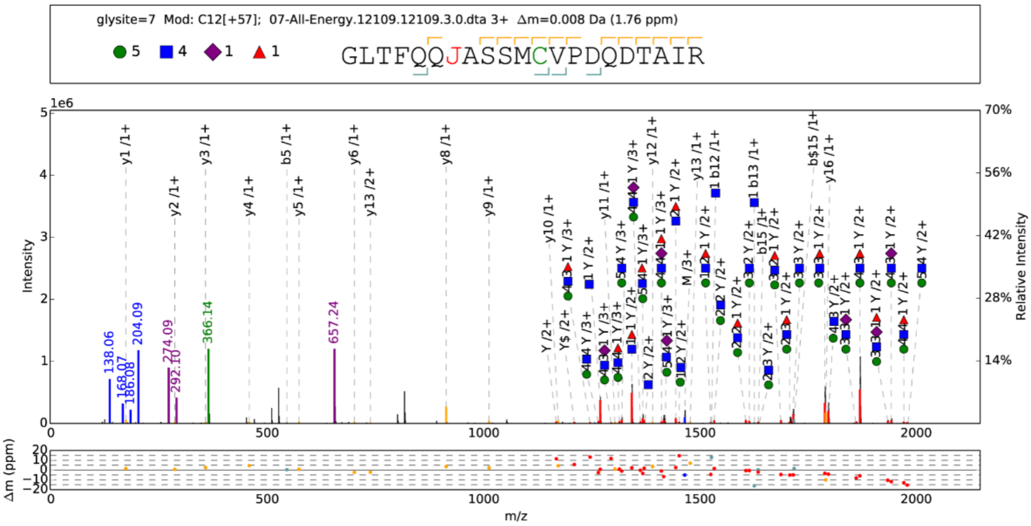

glysite=7 Mod: C12[+57]; 07-All-Energy.12109.12109.3.0.dta 3+ Δm=0.008 Da (1.76 ppm)
GLTFQQJASSMCVPDQDTAIR
Intensity
Relative Intensity
Δm (ppm)
m/z

b2

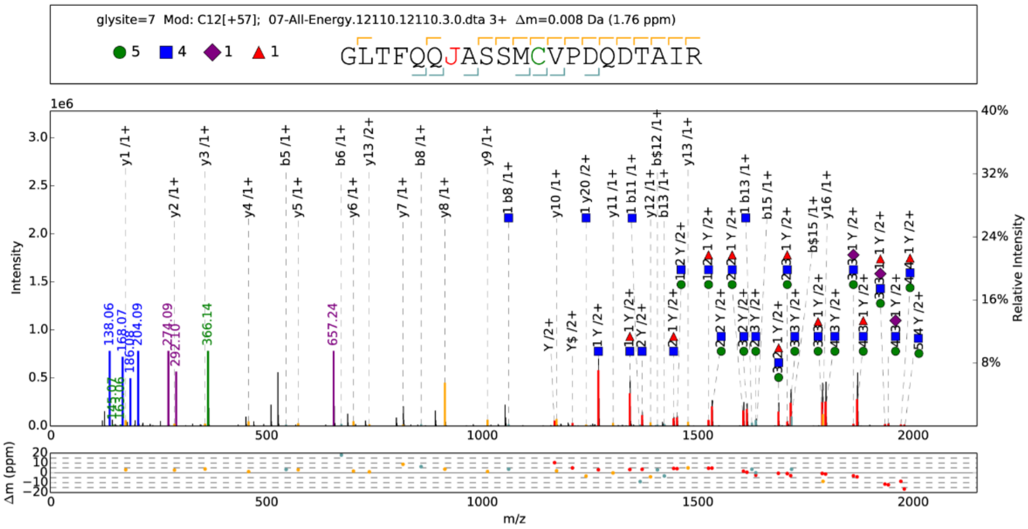

glysite=7 Mod: C12[+57]; 07-All-Energy.12110.12110.3.0.dta 3+ Δm=0.008 Da (1.76 ppm)
GLTFQQJASSMCVPDQDTAIR
Intensity
Relative Intensity
Δm (ppm)
m/z

c2

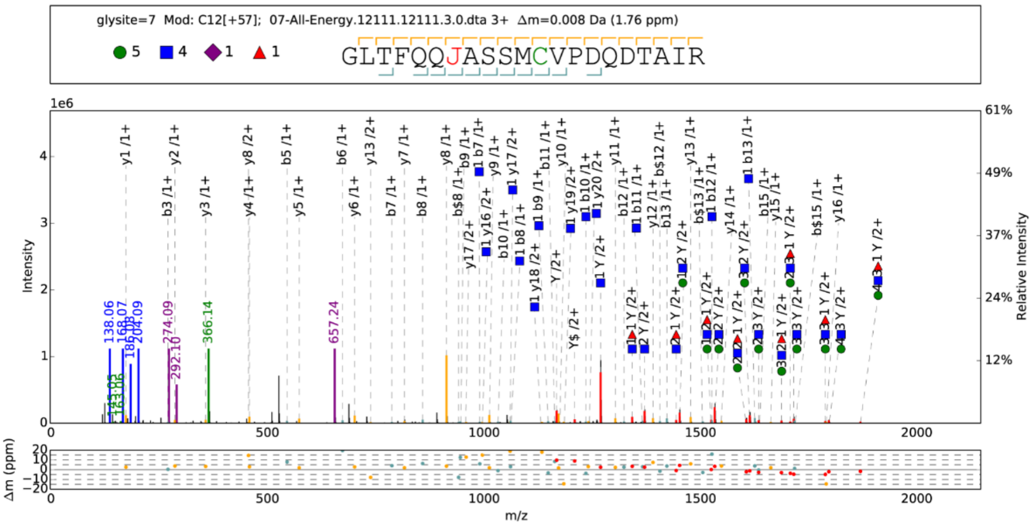

glysite=7 Mod: C12[+57]; 07-All-Energy.12111.12111.3.0.dta 3+ Δm=0.008 Da (1.76 ppm)
GLTFQQJASSMCVPDQDTAIR
Intensity
Relative Intensity
Δm (ppm)
m/z

a3

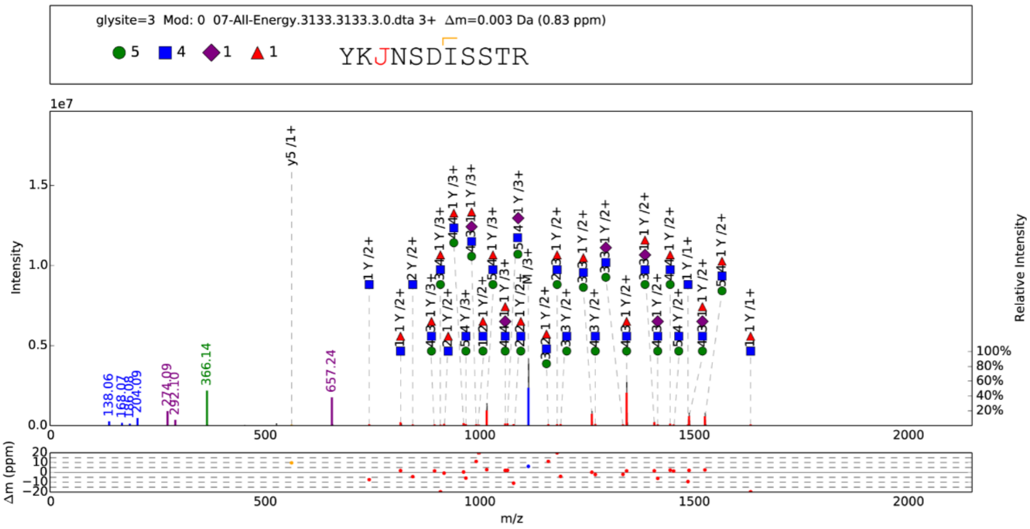
glysite=3 Mod: 0 07-All-Energy.3133.3133.3.0.dta 3+ Δm=0.003 Da (0.83 ppm)
5 4 1 1
YKJNSDISSTR
Intensity
Relative Intensity
Δm (ppm)
m/z

b3

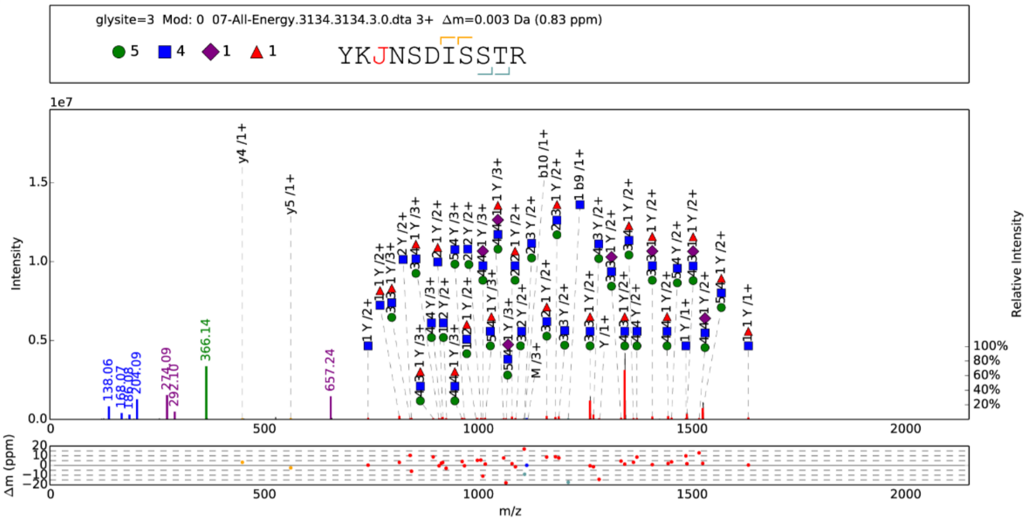
glysite=3 Mod: 0 07-All-Energy.3134.3134.3.0.dta 3+ Δm=0.003 Da (0.83 ppm)
5 4 1 1
YKJNSDISSTR
Intensity
Relative Intensity
Δm (ppm)
m/z

c3

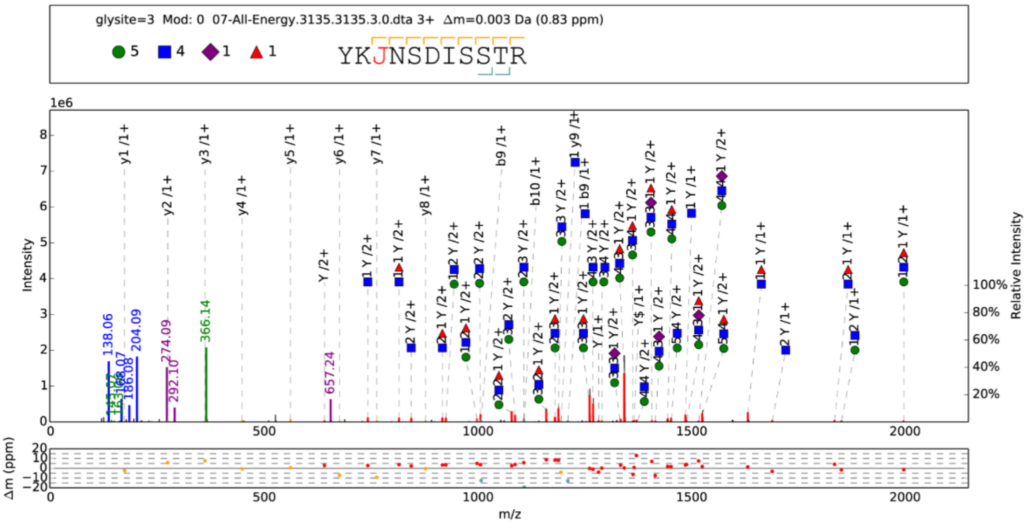
glysite=3 Mod: 0 07-All-Energy.3135.3135.3.0.dta 3+ Δm=0.003 Da (0.83 ppm)
5 4 1 1
YKJNSDISSTR
Intensity
Relative Intensity
Δm (ppm)
m/z

Supplementary Figure 2. Illustration of the different optimum collision energies for different glycopeptides. Three different glycopeptides, each with three HCD-MS/MS spectra obtained under collision energies of 15% (a), 20% (b) and 25% (c) are shown. The design of the annotation in each spectrum is the same as that in Supplementary Figure 1. Although HCD with stepped energies of 20%, 30% and 40% achieved the best overall performance in glycopeptide fragmentation analysis (Supplementary Note 1), it was clear that significant differences existed between the optimum collision energies for different glycopeptides. Three different glycopeptides with the following same glycan composition were selected: Hex × 5 + HexNAc × 4 + NeuAc × 1 + Fucose × 1. The HCD-MS/MS spectra obtained under collision energies of 15%, 20% and 25% for each glycopeptide are shown here. The glycopeptide GLTFQQJASSM produced the most extensive Y ions under a collision energy of 15%, while the glycopeptides GLTFQQJASSMCVPDQDTAIR and YKJNSDISSTR produced most extensive Y ions under collision energies of 20% and 25%, respectively. Based on the findings mentioned above and the manual inspection of thousands of glycopeptide spectra, we concluded that HCD-MS/MS with a collision energy between 15% and 25% is the optimum range for the fragmentation of Y ions, while the optimum range for b/y ions is between 35% and 45%. A more flexible MS/MS collision energy parameter with a user defined range (which is not widely available in the current generation of MS instruments) would improve the performance of

glycopeptide analysis.

Supplementary Figure 3

Example of different glycopeptide identifications reported by pGlyco 2.0 and Byonic

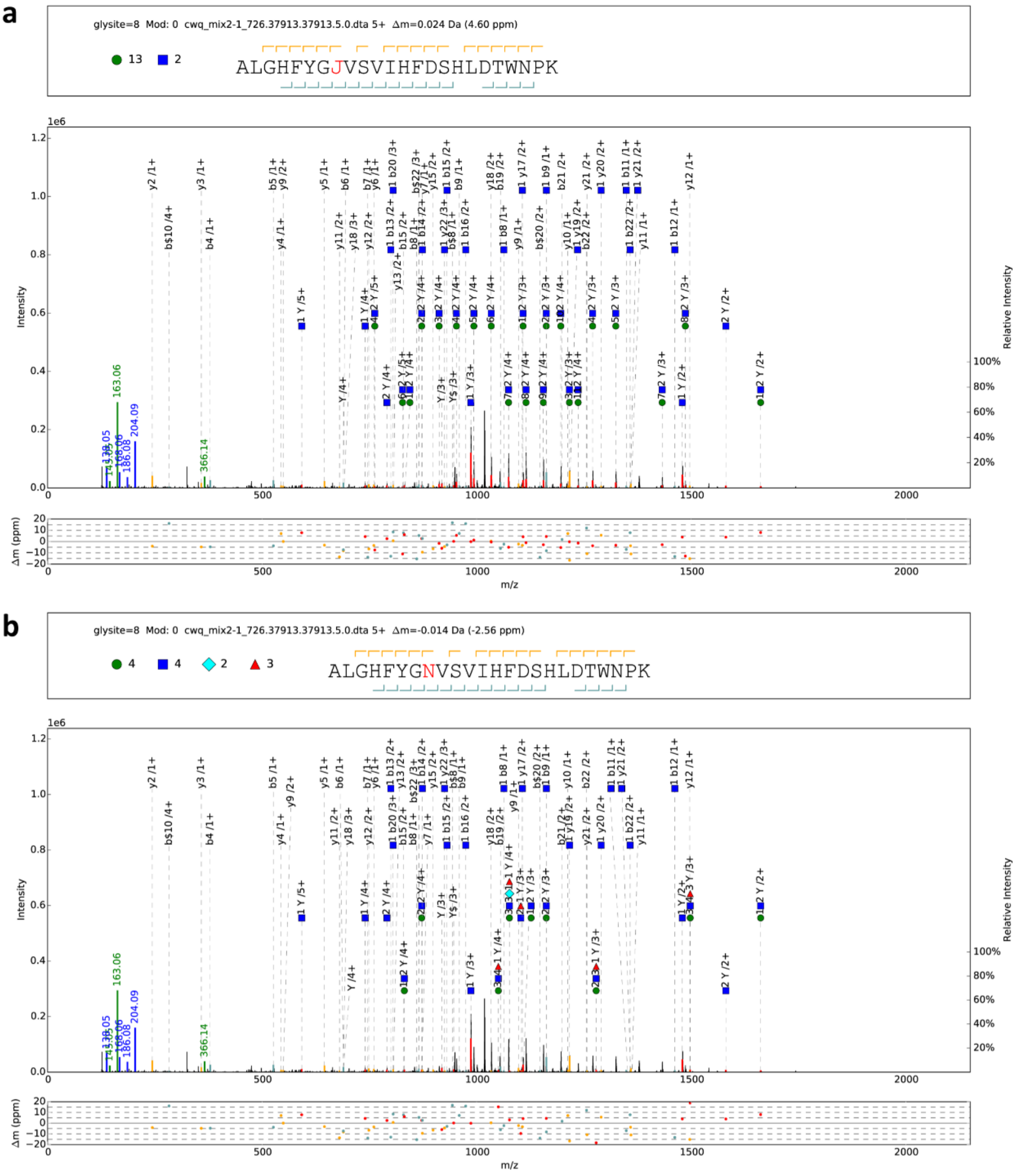

Supplementary Figure 3. Different glycopeptide identifications from pGlyco 2.0 and Byonic for the same spectrum. a) Glycopeptide with a high mannose

content interpreted by pGlyco 2.0. b) Glycopeptide interpreted by Byonic from the same spectrum. The glycan reported by Byonic could not be synthesized by yeast. The design of the annotation in each spectrum is the same as that in Supplementary Figure 1.

Supplementary Figure 4

Retention times of glycopeptides with microheterogeneity

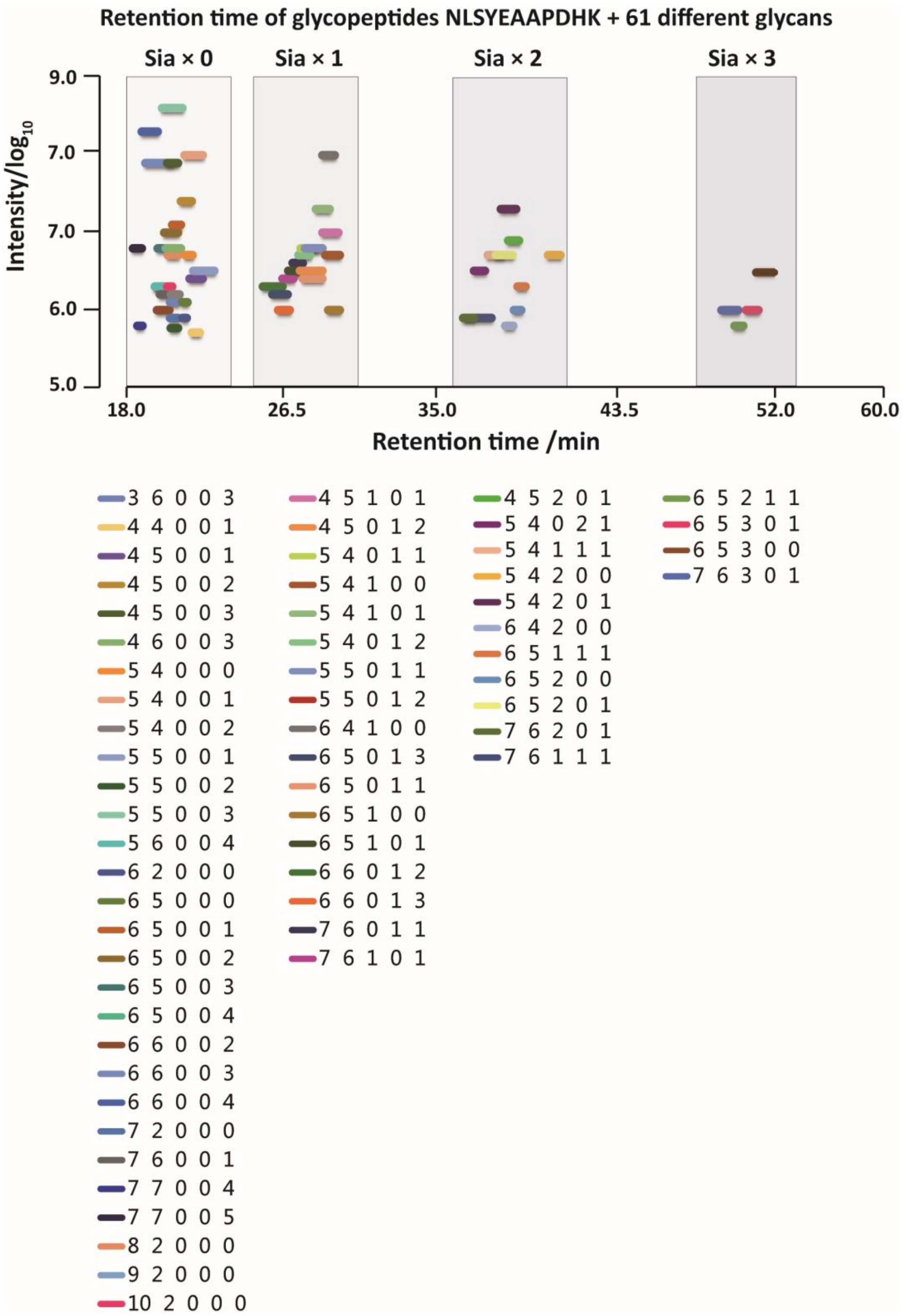

Supplementary Figure 4. Retention times of glycopeptides with microheterogeneity. The retention times of 61 different glycopeptides with the same peptide backbone "NLSYEAAPDHK". The y-axis represents intensity (log 10), the x-axis represents the retention time window, and each color represents a different glycopeptide. "Sia × 0, 1, 2, and 3" indicates that glycopeptides with different numbers of sialic acids (NeuAc or NeuGc) were separated. The glycopeptide compositions are shown at the bottom, and the five digits denote the number of glycans of Hex / HexNAc / NeuAc / NeuGc / fucose attached to the peptide backbone.

Supplementary Figure 5

Analysis of the peak intensity in glycopeptide- and peptide-spectrum matches

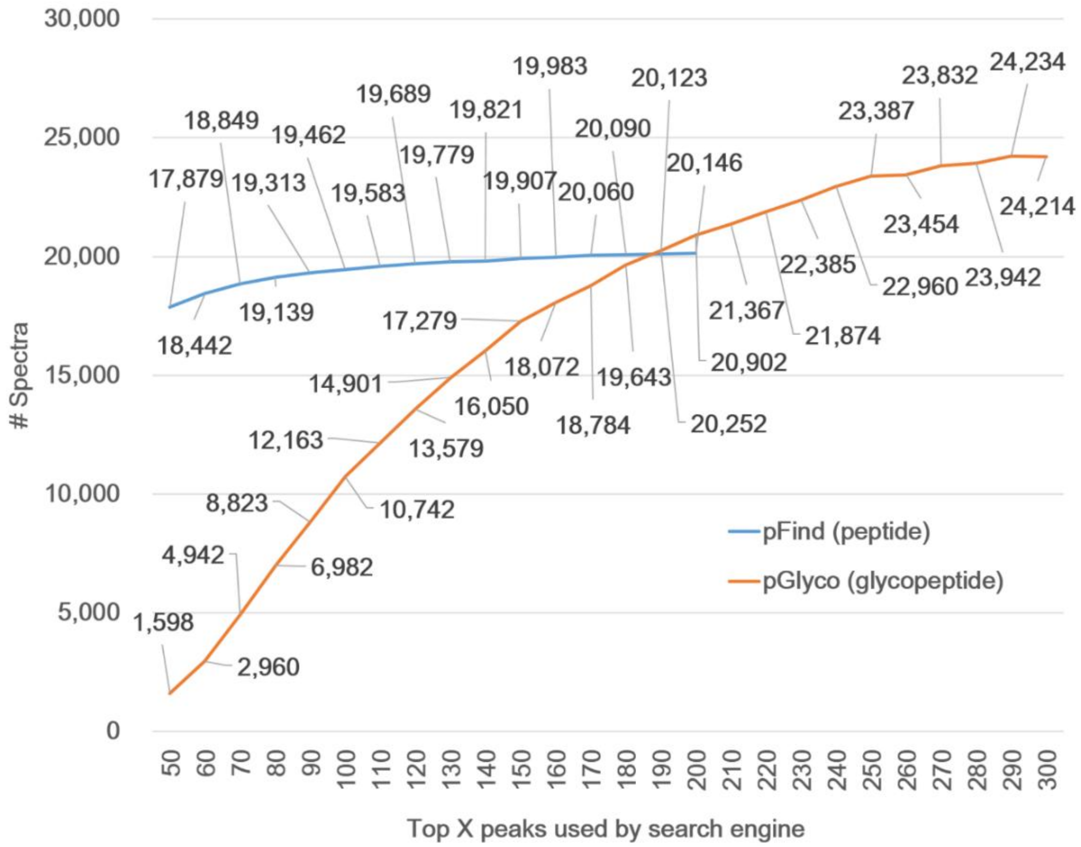

Supplementary Figure 5. Analysis of the peak intensity in glycopeptide- and peptide-spectrum matches. The MS/MS data from mouse kidney were used. Independent database searches for glycopeptides and regular peptides in the same MS/MS data were carried out using pGlyco 2.0 and pFind, respectively (Methods). We used a homemade script to select the top X peaks (50, 60, 70 and 300 in terms of intensity) in each spectrum and compared the database search results for the different peak numbers. For peptide analysis, the number

of spectra identified under the "top 50 peaks" condition was 88.7% of that under the "top 200 peaks" condition (17,879 / 20,146), while for glycopeptide analysis, the number of spectra identified under the "top 50 peaks" condition was only 7.6% of that under the "top 200" condition (1,598 / 20,902), which suggested that the glycopeptide fragments have a much wider dynamic range within each spectrum than the regular peptides do. Many glycopeptide fragments in SCE-HCD-MS/MS have an inherent low intensity. In pGlyco 2.0, we fine-tuned the parameter accordingly and used the top 300 peaks in each spectrum as the default.

Supplementary Figure 6

Examples of chimera spectra from multiple glycopeptides

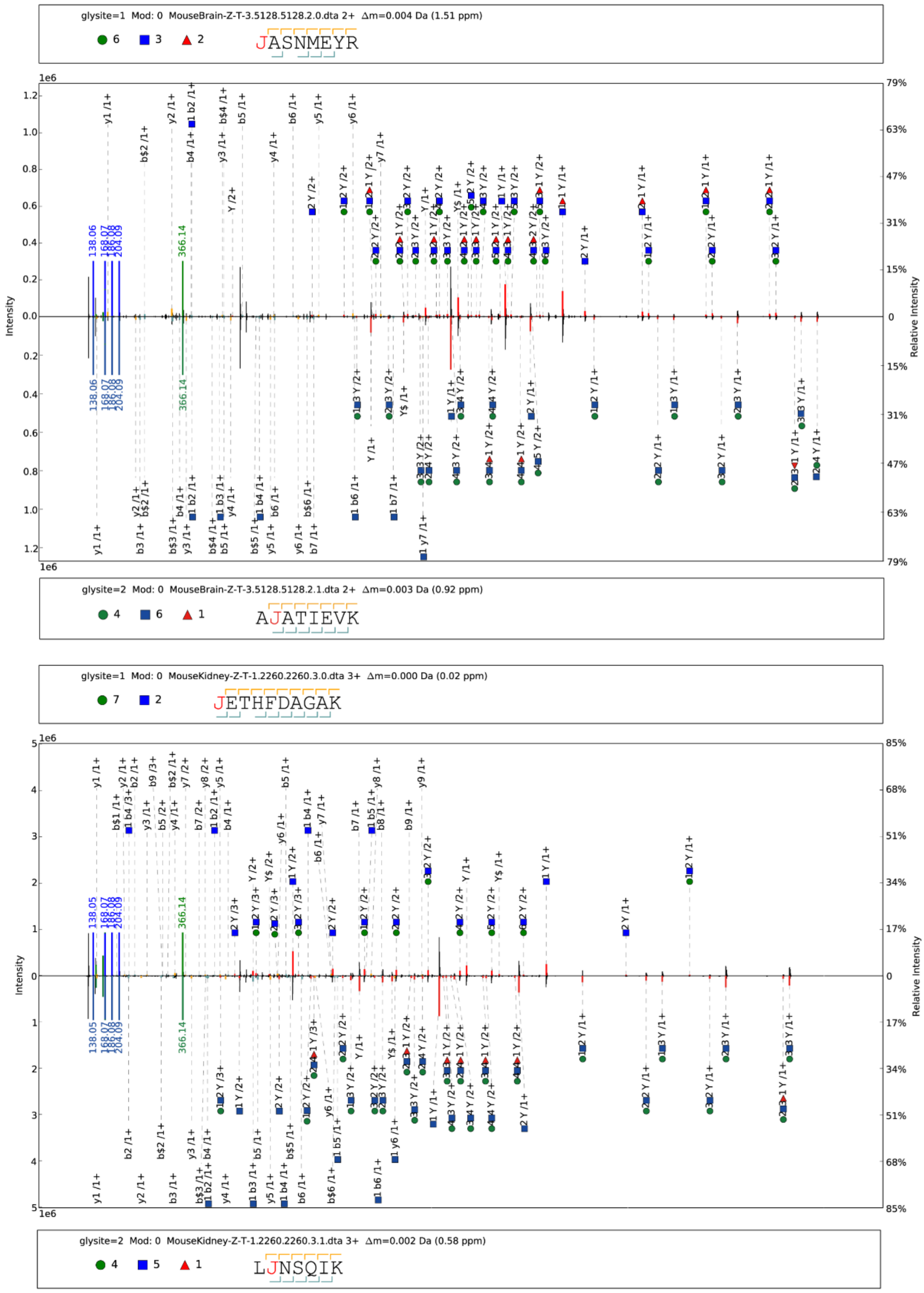

Supplementary Figure 6. Examples of chimera spectra from multiple glycopeptides. Two chimera spectra of multiple glycopeptides are shown: pGlyco 2.0 identified two different glycopeptides in each spectrum, illustrated in the form of a mirrored spectrum annotation. The top and bottom spectra in each figure are the same spectrum with different glycopeptide identifications. The design of the annotation in each spectrum is the same as that in Supplementary Figure 1.

Supplementary Figure 7

MS1 information of chimera spectra from multiple glycopeptides

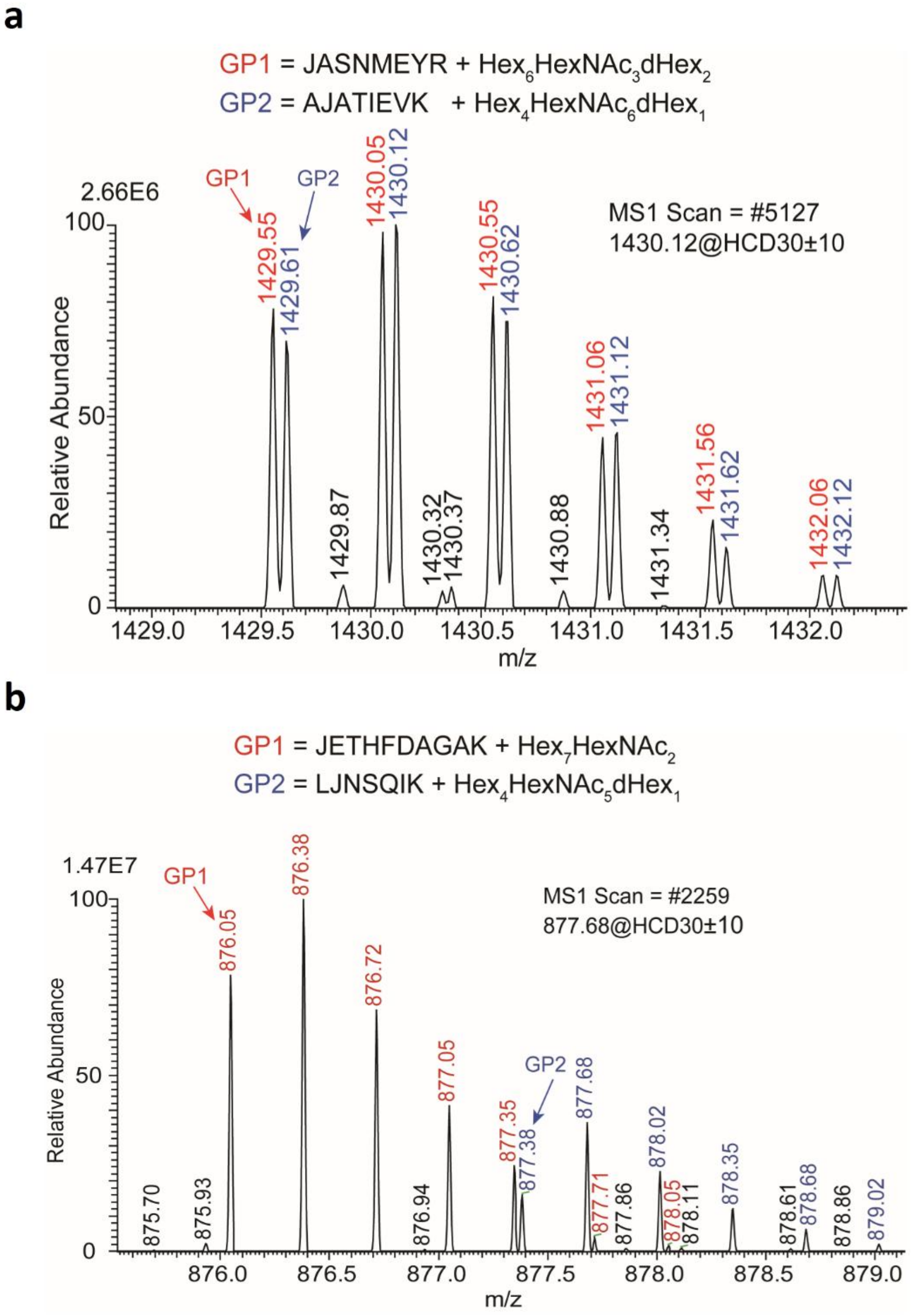

Supplementary Figure 7. MS1 data of two example chimera spectra from the multiple glycopeptides shown in Supplementary Figure 6.

Supplementary Figure 8

Correlation analysis of glycopeptide profiling among all LC-MS runs

		Brain					Heart					Kidney					Liver					Lung				
		R1	R2	R3	R4	R5	R1	R2	R3	R4	R5	R1	R2	R3	R4	R5	R1	R2	R3	R4	R5	R1	R2	R3	R4	R5
Brain	R1	1	.89	.91	.95	.75	-.01	-.01	-.01	-.01	-.01	-.02	-.02	-.02	-.02	-.02	-.01	-.01	-.01	-.01	-.01	-.01	-.01	-.02	-.01	-.01
	R2	.89	1	.91	.90	.76	-.01	0	0	0	0	-.02	-.02	-.02	-.02	-.02	-.01	-.01	-.01	-.01	-.01	-.01	-.01	-.01	-.01	-.01
	R3	.91	.91	1	.93	.74	-.01	-.01	-.01	-.01	-.01	-.02	-.02	-.02	-.02	-.02	-.01	-.01	-.01	-.01	-.01	-.01	-.01	-.01	-.01	-.01
	R4	.95	.90	.93	1	.74	-.01	-.01	-.01	-.01	-.01	-.02	-.02	-.02	-.02	-.02	-.01	-.01	-.01	-.01	-.01	-.01	-.01	-.01	-.01	-.01
	R5	.75	.76	.74	.74	1	-.01	-.01	-.01	0	0	-.02	-.02	-.02	-.02	-.02	0	0	-.01	0	0	-.01	-.01	-.01	-.01	-.01
Heart	R1	-.01	-.01	-.01	-.01	-.01	1	.83	.86	.86	.83	.02	.02	.01	.01	.02	.05	.05	.05	.05	.05	.29	.36	.28	.29	.30
	R2	-.01	0	-.01	-.01	-.01	.83	1	.95	.96	.97	.02	.02	.01	.01	.02	.03	.02	.03	.02	.02	.42	.50	.38	.41	.44
	R3	-.01	0	-.01	-.01	-.01	.86	.95	1	.98	.95	.01	.02	0	.01	.02	.01	.01	.02	.01	.01	.39	.47	.36	.38	.42
	R4	-.01	0	-.01	-.01	0	.86	.96	.98	1	.97	.01	.02	.01	.01	.02	.02	.02	.02	.02	.01	.37	.45	.35	.37	.40
	R5	-.01	0	-.01	-.01	0	.83	.97	.95	.97	1	.02	.02	.01	.01	.02	.01	.01	.02	.01	.01	.40	.48	.36	.39	.43
Kidney	R1	-.02	-.02	-.02	-.02	-.02	.02	.02	.01	.01	.02	1	.93	.92	.93	.93	.04	.04	.05	.05	.04	.03	.04	.03	.03	.03
	R2	-.02	-.02	-.02	-.02	-.02	.02	.02	.02	.02	.02	.93	1	.95	.94	.92	.04	.04	.05	.06	.04	.03	.03	.03	.03	.03
	R3	-.02	-.02	-.02	-.02	-.02	.01	.01	0	.01	.01	.92	.95	1	.90	.91	.05	.05	.05	.06	.04	.02	.02	.02	.02	.03
	R4	-.02	-.02	-.02	-.02	-.02	.01	.01	.01	.01	.01	.93	.94	.90	1	.92	.04	.05	.05	.05	.04	.02	.03	.03	.03	.03
	R5	-.02	-.02	-.02	-.02	-.02	.02	.02	.02	.02	.02	.93	.92	.91	.92	1	.03	.03	.03	.04	.03	.03	.03	.03	.03	.03
Liver	R1	-.01	-.01	-.01	-.01	0	.05	.03	.01	.02	.01	.04	.04	.05	.04	.03	1	.94	.95	.93	.93	.07	.12	.12	.10	.10
	R2	-.01	-.01	-.01	-.01	0	.05	.02	.01	.02	.01	.04	.04	.05	.05	.03	.94	1	.93	.94	.93	.07	.12	.11	.09	.10
	R3	-.01	-.01	-.01	-.01	-.01	.05	.03	.02	.02	.02	.05	.05	.05	.05	.03	.95	.93	1	.94	.93	.08	.13	.11	.10	.11
	R4	-.01	-.01	-.01	-.01	0	.05	.02	.01	.02	.01	.05	.06	.06	.05	.04	.93	.94	.94	1	.93	.07	.11	.10	.09	.09
	R5	-.01	-.01	-.01	-.01	0	.05	.02	.01	.01	.01	0.4	.04	.04	.04	.03	.93	.93	.93	.93	1	.07	.10	.10	.08	.08
Lung	R1	-.01	-.01	-.01	-.01	-.01	.29	.42	.39	.37	.40	.03	.03	.02	.02	.03	.07	.07	.08	.07	.07	1	.91	.91	.93	.94
	R2	-.01	-.01	-.01	-.01	-.01	.36	.50	.47	.45	.48	.04	.03	.02	.03	.03	.12	.12	.13	.11	.10	.91	1	.89	.91	.91
	R3	-.01	-.01	-.01	-.01	-.01	.28	.38	.36	.35	.36	.03	.03	.02	.03	.03	.12	.11	11	.10	.10	.91	.89	1	.94	.91
	R4	-.01	-.01	-.01	-.01	-.01	.29	.41	.38	.37	.39	.03	.03	.02	.03	.03	.10	.09	.10	.09	.08	.93	.91	.94	1	.92
	R5	-.01	-.01	-.01	-.01	-.01	.30	.44	.42	.40	.43	.03	.03	.03	.03	.03	.10	.10	.11	.09	.08	.94	.91	.91	.92	1

Supplementary Figure 8. Correlation analysis of glycopeptide profiling among all LC-MS runs. Each tissue was analyzed five times, and the correlation of all 25 LC-MS runs is shown. The values were calculated using Pearson correlation coefficient analysis based on the spectrum count values of all identified glycopeptides in each LC-MS run. R1 to R5 means five replicate runs for each mouse tissue.

Supplementary Figure 9

NeuAc and NeuGc profiling through DMB labeling and UHPLC analysis

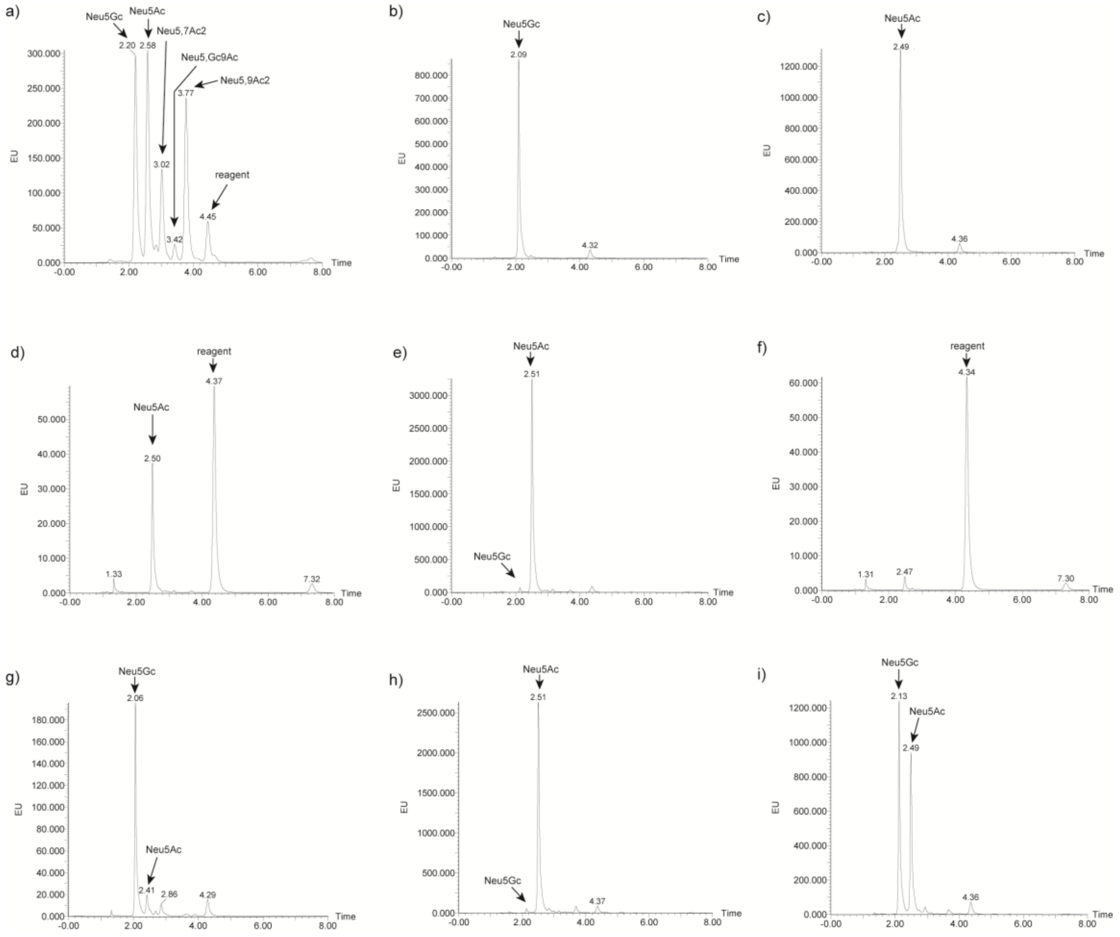

Supplementary Figure 9. Abundance of NeuAc and NeuGc in different samples. (a) Reference panel. (b) Standard NeuGc. (c) Standard NeuAc. (d) Human IgG. (e) Bovine fetuin. (f) Ovalbumin. (g) Mouse liver. (h) Mouse brain. (i) Mouse kidney. The sialic acids from glycoproteins were released and specifically labeled using a LudgerTag™ DMB (1,2-diamino-4,5-methylenedioxybenzene.2HCl) Kit. Then, the DMB-labeled sialic acids were identified and relatively quantitated using reversed-phase chromatography.

First, a DMB-labeled sialic reference panel (containing Neu5Ac, Neu5Gc, Neu5,7Ac2, Neu5,Gc9Ac and Neu5,9Ac2) and NeuAc and NeuGc standard monosaccharide were analyzed as standards (Supplementary Fig. 9-a to 9-c). Then, human IgG, which only contains Neu5Ac, and bovine fetuin, which contains predominantly Neu5Ac and a small amount of NeuGc (2-3%), were analyzed as positive controls (Supplementary Fig. 9-d, e). Ovalbumin, which contains only trace amounts of sialic acids, was analyzed as a negative control (Supplementary Fig. 9-f). The analysis results were highly consistent with existing knowledge. Finally, DMB-labeled sialic acids from mouse liver, brain and kidney were analyzed to determine the relative amounts of Neu5Ac and Neu5Gc in each tissue. Trace amounts of NeuAc were detected in the liver (Supplementary Fig. 9-g), while the opposite distribution of sialic acids was observed in the brain (Supplementary Fig. 9-h). Meanwhile, the amounts of NeuGc and NeuAc in the kidney were similar (Supplementary Fig. 9-i). The above analysis results on the abundance of NeuAc and NeuGc in mouse tissues were consistent with our glycopeptide data obtained using pGlyco 2.0.

Supplementary Figure 10

Comparison between glycopeptide spectra with and without stepped energy

a1

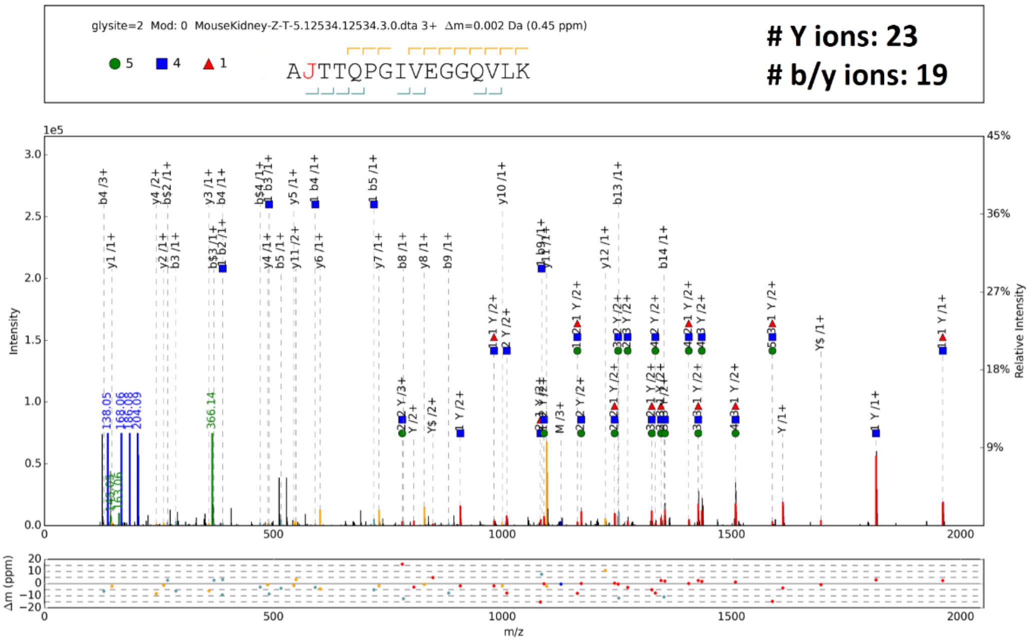

b1

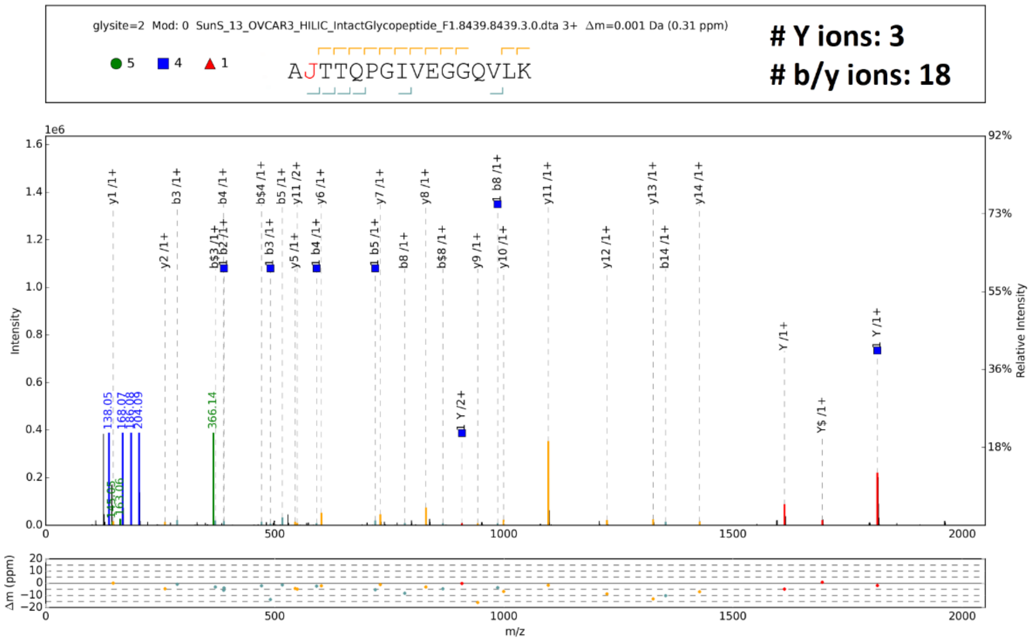

a2

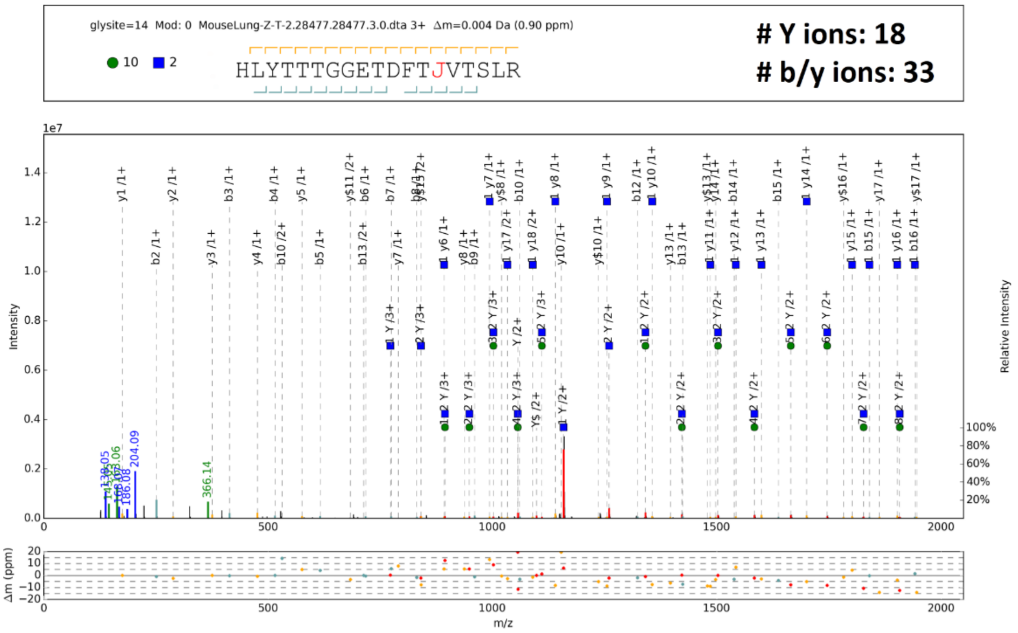

b2

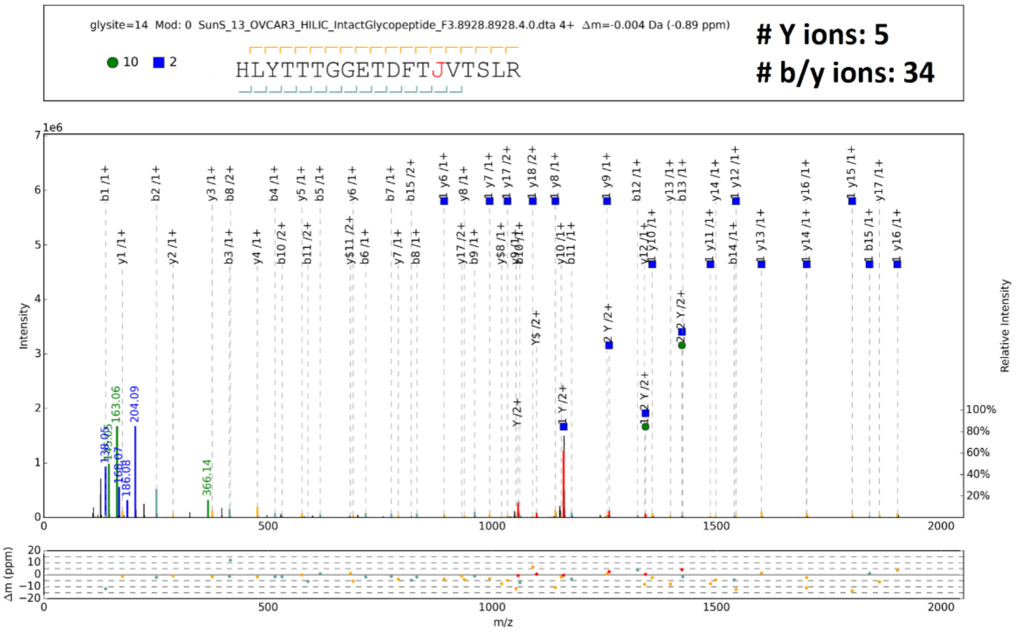

Supplementary Figure 10. Comparison of glycopeptide spectra obtained with (in this work) and without (previously reported work) stepped energy. For each spectrum pair, two spectra from the same glycopeptide are shown. (a) Spectrum from our data obtained using SCE-HCD-MS/MS. (b) Spectrum from the reference obtained using single-energy HCD-MS/MS. In each case, the

spectrum with the highest score for the glycopeptide was selected. The design of the annotation in each spectrum is the same as that in Supplementary Figure 1. The numbers of Y ions and b/y ions in each spectrum are shown in the upper right corner.

Supplementary Figure 11

Venn diagram of glycopeptide identification results from pGlyco 2.0 and GPQuest for the same MS/MS data.

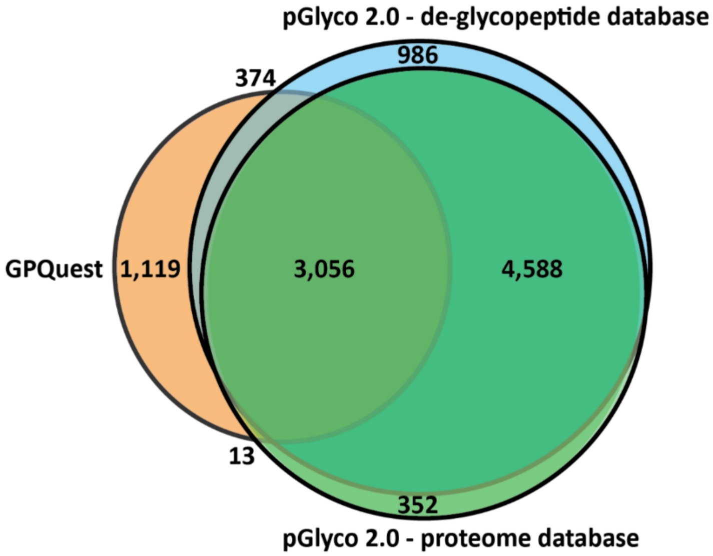

Supplementary Figure 11. Venn diagram of glycopeptide identification results from pGlyco 2.0 and GPQuest for the same MS/MS data. “GPQuest” represents previously published data obtained using GPQuest; “pGlyco 2.0 – deglycopeptide database” represents glycopeptide identification by pGlyco 2.0 for the deglycopeptide database reported by GPQuest; “pGlyco 2.0 – proteome database” represents glycopeptide identification by pGlyco 2.0 for the complete human proteome database.

Supplementary Figure 12

Comparison with previously reported glycosylation site data in mouse tissues

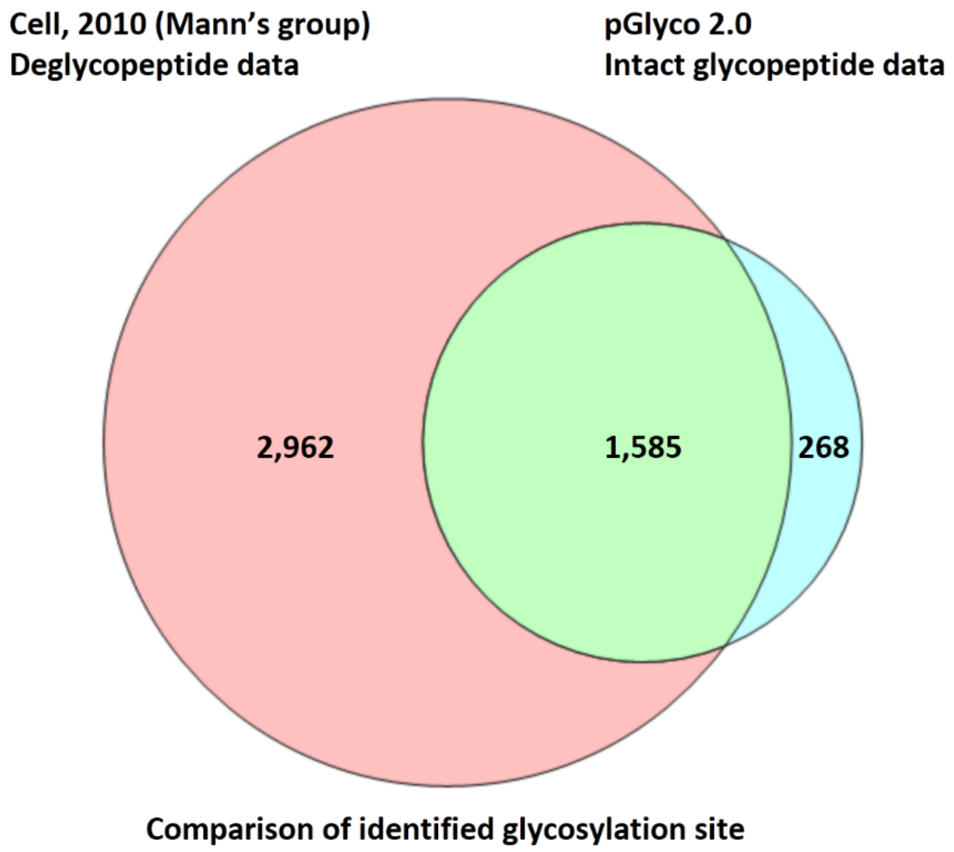

Supplementary Figure 12. Comparison of all identified glycosylation sites in four mouse tissues (brain, heart, kidney, and liver). In our data, 85% of the identified glycosylation sites were reported in the reference. The targets in the glycosylation site data from the reference were deglycopeptides that did not have site-specific glycan information, while the targets in our data were intact glycopeptides that had abundant site-specific glycan information and lower

abundance and lower ionization efficiency than those of deglycopeptides. In addition, multiple glyco-enrichments and protein-digestion enzymes were used in the reference, while we only used a single glyco-enrichment and a single protein-digestion enzyme, which partially explains the difference in the size of glycosylation site data.

Supplementary Figure 13

N- and O-glycosylation analyses of asialofetuin

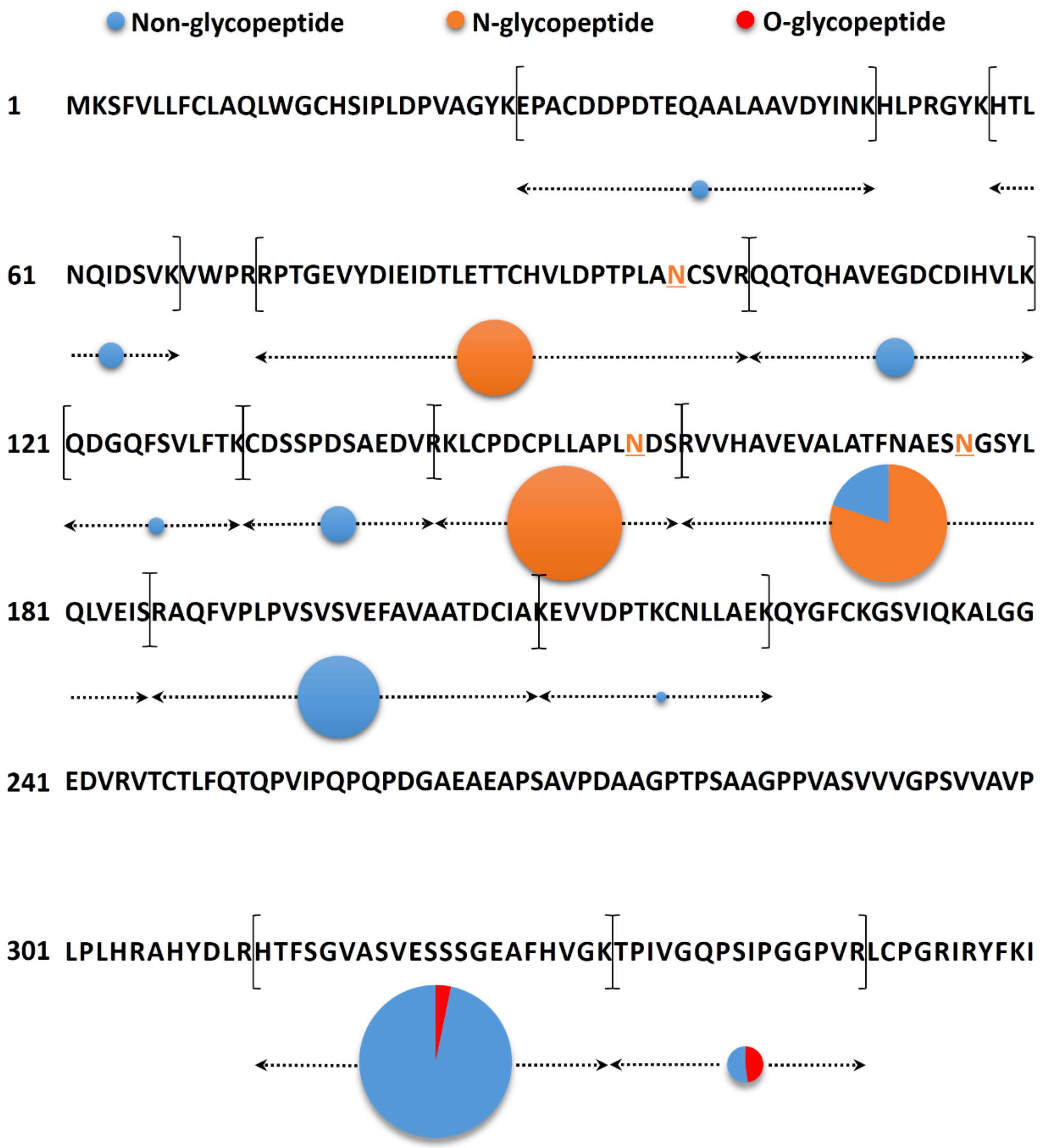

Supplementary Figure 13. N- and O-glycosylation analyses of asialofetuin. Glyco-enriched asialofetuin was analyzed using SCE-HCD-MS/MS, and glycopeptides and regular peptides were then analyzed using pGlyco 2.0 and pFind, respectively. The theoretical sequence of asialofetuin starts with a

number, for example, the line that starts with “1” shows the first sixty amino acids of asialofetuin. The identified glycopeptides and regular peptides are shown in the theoretical sequence: the dotted line with arrows on either side corresponds to the sequence of the peptide backbone; the identified non-glycopeptides, N-glycopeptides and O-glycopeptides are shown in blue, orange and red, respectively; and the area of the circle is proportional to the number of identified MS/MS spectra.

Supplementary Table 1

Tissue specific, high-abundance glycoproteins in five mouse tissues

Protein Accession	Protein Description	Brain	Heart	Kidney	Liver	Lung
P43006	Excitatory amino acid transporter 2	180 (5.4%)	0 (0.0%)	0 (0.0%)	1 (0.0%)	0 (0.0%)
P01831	Thy-1 membrane glycoprotein	117 (3.5%)	2 (0.2%)	2 (0.1%)	0 (0.0%)	2 (0.1%)
Q62277	Synaptophysin	84 (2.5%)	0 (0.0%)	0 (0.0%)	0 (0.0%)	0 (0.0%)
P14231	Sodium/potassium-transporting ATPase subunit beta-2	83 (2.5%)	1 (0.1%)	0 (0.0%)	1 (0.1%)	2 (0.1%)
P12960	Contactin-1	71 (2.1%)	0 (0.0%)	0 (0.0%)	0 (0.0%)	0 (0.0%)
Q60675	Laminin subunit alpha-2	4 (0.1%)	97 (10%)	4 (0.1%)	0 (0.0%)	18 (0.7%)
A2ARV4	Low-density lipoprotein receptor-related protein 2	0 (0.0%)	0 (0.0%)	370 (9.5%)	0 (0.0%)	4 (0.2%)
O88338	Cadherin-16	0 (0.0%)	0 (0.0%)	154 (3.9%)	0 (0.0%)	0 (0.0%)
Q9JLB4	Cubilin	0 (0.0%)	0 (0.0%)	112 (2.9%)	0 (0.0%)	0 (0.0%)
P28825	Meprin A subunit alpha	0 (0.0%)	0 (0.0%)	107 (2.7%)	0 (0.0%)	0 (0.0%)
Q60928	Gamma-glutamyltranspeptidase 1	1 (0.0%)	0 (0.0%)	88 (2.2%)	0 (0.0%)	0 (0.0%)
Q01279	Epidermal growth factor receptor	1 (0.0%)	0 (0.0%)	2 (0.1%)	38 (1.6%)	1 (0.0%)
P31809	Carcinoembryonic antigen-related cell adhesion molecule 1	0 (0.0%)	1 (0.1%)	3 (0.1%)	29 (1.3%)	3 (0.1%)
Q63880	Carboxylesterase 3A	0	0	0	16	0

		(0.0%)	(0.0%)	(0.0%)	(0.7%)	(0.0%)
**Q8QZR3**	**Pyrethroid hydrolase Ces2a**	**0 (0.0%)**	**0 (0.0%)**	**0 (0.0%)**	**14 (0.6%)**	**0 (0.0%)**
**Q62452**	**UDP-glucuronosyltransferase 1-9**	**0 (0.0%)**	**0 (0.0%)**	**0 (0.0%)**	**12 (0.5%)**	**0 (0.0%)**
**P13597**	**Intercellular adhesion molecule 1**	**0 (0.0%)**	**0 (0.0%)**	**5 (0.1%)**	**2 (0.1%)**	**51 (2.0%)**
**A2ARA8**	**Integrin alpha-8**	**0 (0.0%)**	**0 (0.0%)**	**0 (0.0%)**	**0 (0.0%)**	**20 (0.8%)**
**P15306**	**Thrombomodulin**	**0 (0.0%)**	**1 (0.1%)**	**0 (0.0%)**	**0 (0.0%)**	**15 (0.6%)**
**P35441**	**Thrombospondin-1**	**0 (0.0%)**	**0 (0.0%)**	**1 (0.0%)**	**0 (0.0%)**	**14 (0.6%)**
**Q8BHC0**	**Lymphatic vessel endothelial hyaluronic acid receptor 1**	**0 (0.0%)**	**0 (0.0%)**	**0 (0.0%)**	**0 (0.0%)**	**13 (0.5%)**

Supplementary Table 1. Tissue specific, high-abundance glycoproteins in five mouse tissues. Top tissue-specific glycoproteins in each mouse tissue are shown in the table. Here, "tissue-specific" means that the relative abundance of the unique glycopeptides identified in one tissue was at least ten times higher than that in the four other tissues. The abundance was ranked using the number of unique glycopeptides. The number in each grid shows the number and the relative abundance of the unique glycopeptides of the glycoprotein in each tissue.

Supplementary Note 1:

Energy-resolved fragmentation analysis for glycopeptides

To systematically study the glycopeptide fragmentation behavior, we analyzed the glycopeptides in a mixture of five standard glycoproteins under diverse MS/MS conditions on an Orbitrap Fusion Tribrid (Thermo Scientific). The parameters included the following: MS1: scan range (m/z) = 700~2,000; resolution = 120,000; AGC target = 500,000; maximum injection time = 100 ms; included charge state = +2 ~ +6; dynamic exclusion after n time, n = 1; dynamic exclusion duration = 15 s; each selected precursor was subject to 21 different MS/MS analyses, including collision energies for CID-MS/MS = 10%, 15%, 20%, 25%, 30%, 35%, 40%, 45%, and 50% and collision energies for HCD-MS/MS = 10%, 15%, 20%, 25%, 30%, 35%, 40%, 45%, and 50%; ETD/ETcid/EThcD-MS/MS were set in the default methods; isolation mode: quadrupole; isolation window = 2; detector type = Orbitrap; resolution = 15,000; AGC target = 500,000; maximum injection time = 100 ms; and microscan = 1.

We first analyzed the primary fragment ions in high-quality glycopeptide spectra. The interpretation of an HCD-MS/MS spectra was used as an example: 9 single-energy HCD-MS/MS spectra from the same precursor were merged

into a nine-energy spectrum named "AllCE" (All Collisional Energy). We manually verified all results and obtained 166 high-quality GPSMs.

The primary fragment ions of the glycopeptides were classified into ten categories:

1) Y ions, including ${}^{0,2}X_0$ ions, which we named Y$ ($ represents cross-ring fragmentation) in pGlyco 2.0 for simplification;

2) b/y ions of the naked peptide backbone;

3) b$/y$ ions and b/y ions of the naked peptide backbone with a cross-ring fragment;

4) b/y + 01000 ions (b/y + HexNAc × 1);

5) b/y + 02000 ions (b/y + HexNAc × 2);

6) b/y + 12000 ions (b/y + Hex × 1 + HexNAc × 2);

7) b/y + 22000 ions (b/y + Hex × 2 + HexNAc × 2);

8) b/y + 32000 ions (b/y + Hex × 3 + HexNAc × 2);

9) b/y + 01001 ions (b/y + HexNAc × 1 + dHex × 1);

10) b/y + 02001 ions (b/y + HexNAc × 2 + dHex × 2);

The number of occurrences and the relative and absolute intensity of each ion category in all "AllCE" spectra were statistically analyzed, as shown in Supplementary Note 1-1. The most abundant ion categories were Y ions, b/y

ions, b/y + 01000 ions and b$/y$ ions. These four kinds of ions were used in the following analysis.

a

b

c

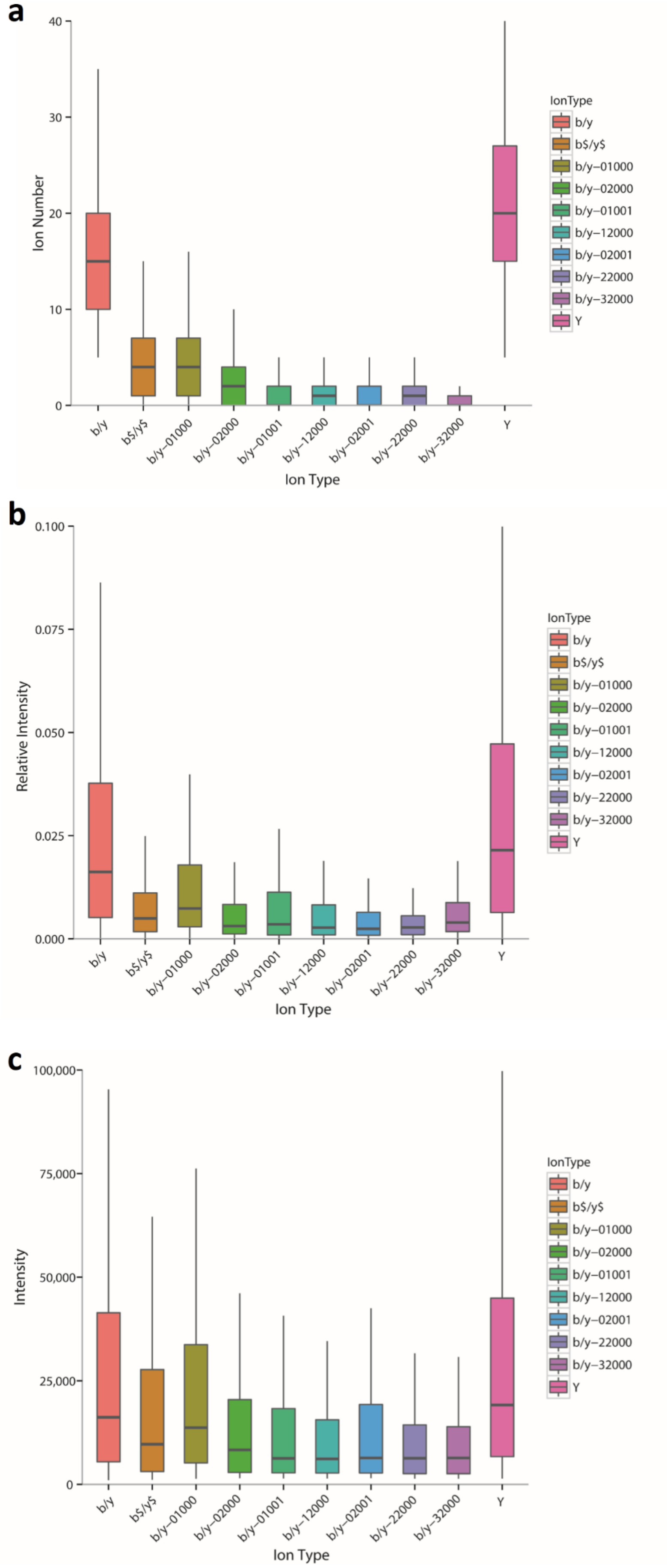

40
30
20
10
0
Ion Number
Ion Type
IonType
b/y
b$/y$
b/y−01000
b/y−02000
b/y−01001
b/y−12000
b/y−02001
b/y−22000
b/y−32000
Y
0.100
0.075
0.050
0.025
0.000
Relative Intensity
100,000
75,000
50,000
25,000
0
Intensity

1

Supplementary Note 1-1. Analysis of 10 different kinds of fragment ions of glycopeptides in 166 high-quality all-energy-merged HCD-MS/MS spectra. (a) Distribution of the ion occurrence. (b) Distribution of the relative ion intensity. (c) Distribution of the absolute ion intensity.

Next, we investigated the distribution of fragment ions in a single spectrum. We compared the theoretical ion coverage of HCD/CID/ETD-MS/MS spectra at each energy with that in "AllCE" spectra. To compare the fragment ion occurrence of glycopeptides with different peptide lengths or glycan sizes, we used the ion ratio, which was the number of matched fragment ions normalized by the number of theoretical fragment ions:

Ion ratio = # matched fragment ions / # theoretical fragment ions

The analysis of HCD-MS/MS spectra is shown in Supplementary Note 1-2a. Y ions were the preferred fragment ions in lower-energy HCD-MS/MS (HCD with energy between 15% and 25%). The ion ratio of the Y ions peaked at an HCD with an energy of 20%. As the energy increased, the Y ions gradually over-fragmented and decreased, while the fragment ions from peptide backbones increased. The ion ratio of peptide backbone fragment ions peaked at an HCD with an energy 40%. Further increase in the energy to 50% decreased the ion ratios of both the Y ions and peptide backbone fragment ions. It is very difficult to find a single energy that can provide abundant information for both the glycan and the peptide of an intact glycopeptide.

The distribution of fragment ions in CID-MS/MS spectra is shown in Supplementary Note 1-2b: the difference between the glycopeptide spectra obtained at different collision energies for CID-MS/MS was subtle.

As the energy increased, the fragmentation usually started at an energy of 20% or 25%. After that, the fragmentation behavior stabilized regardless of the energy. Compared with HCD-MS/MS, CID-MS/MS only provided a tiny portion of fragment ions from the peptide backbone.

Different ETD-MS/MS methods, including ETD/ETciD/EThcD-MS/MS, did not perform well in our analysis, as demonstrated in Supplementary Note 1-2c and 1-2d: ETD/ETciD-MS/MS generated only a few fragment ions, while EThcD-MS/MS generated less fragment ions than HCD-MS/MS.

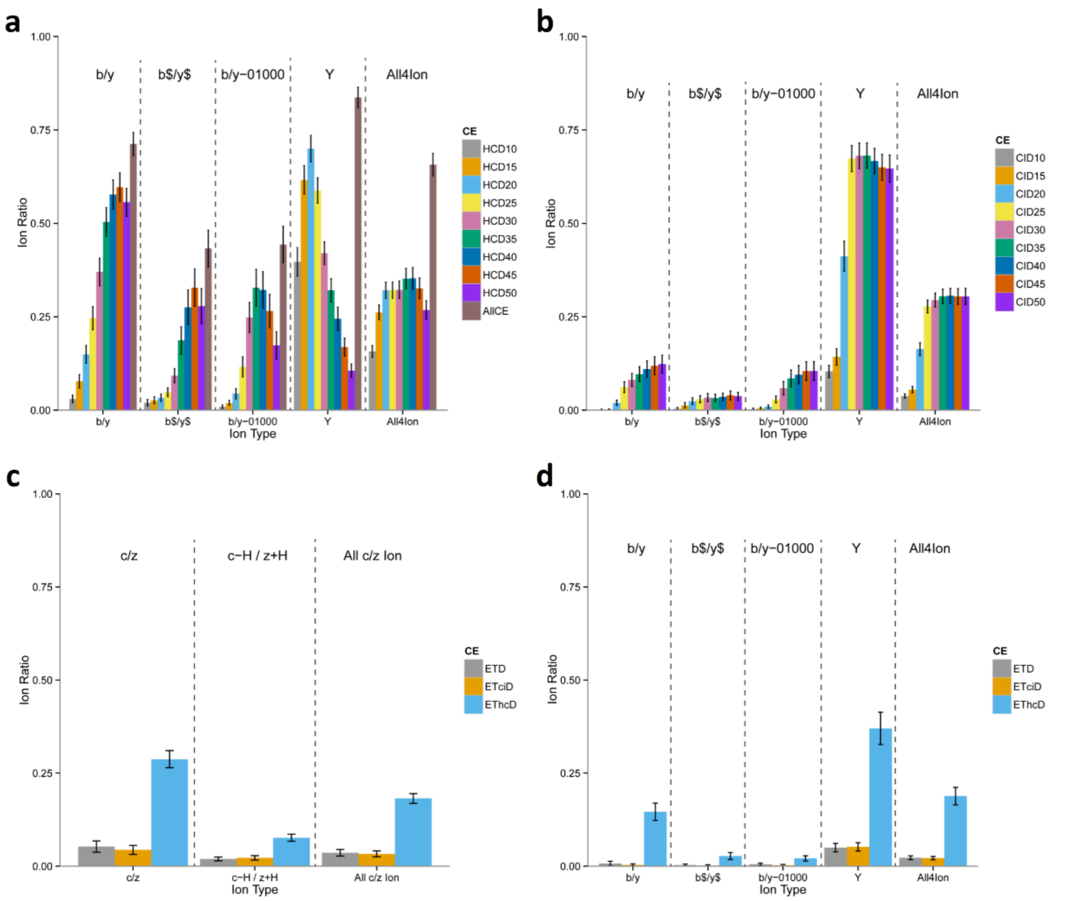

Supplementary Note 1-2. Comparison of the normalized fragment ion coverage in glycopeptide spectra. The normalized matched fragment ion coverage of each ion type is illustrated. (a) Nine single-energy and one merged all-energy HCD-MS/MS spectra. (b) Nine single-energy CID-MS/MS spectra. (c) Different modes of ETD-MS/MS spectra, coverage of c/z fragment ions. (d) Different modes of ETD-MS/MS spectra, coverage of Y ions and b/y fragment ions.

Based on the analysis of HCD/CID/ETD-MS/MS, we concluded that combined HCD-MS/MS with different collision energies or SCE HCD-MS/MS should be the method of choice. As Orbitrap Fusion or Q Exactive instruments only provide 3 SCE instrument configurations for users, we simulated 16 possible configurations of SCE-HCD-MS/MS spectra (Supplementary Fig. 3). Three out of 16 types of SCE-HCD-MS/MS (HCD-15-30-45, HCD-20-30-40 and HCD 20-35-50) were outstanding and showed similar performances to that of the "AllCE" simulation at the ion ratios of both Y ions and peptide backbone fragment ions. By manually verifying the three SCE conditions, HCD-20-30-40 was selected as the preferred condition for glycopeptide fragmentation (Supplementary Note 1-3). In summary, we fine-tuned a high-throughput fragmentation method that can achieve the best performance in a single spectrum.

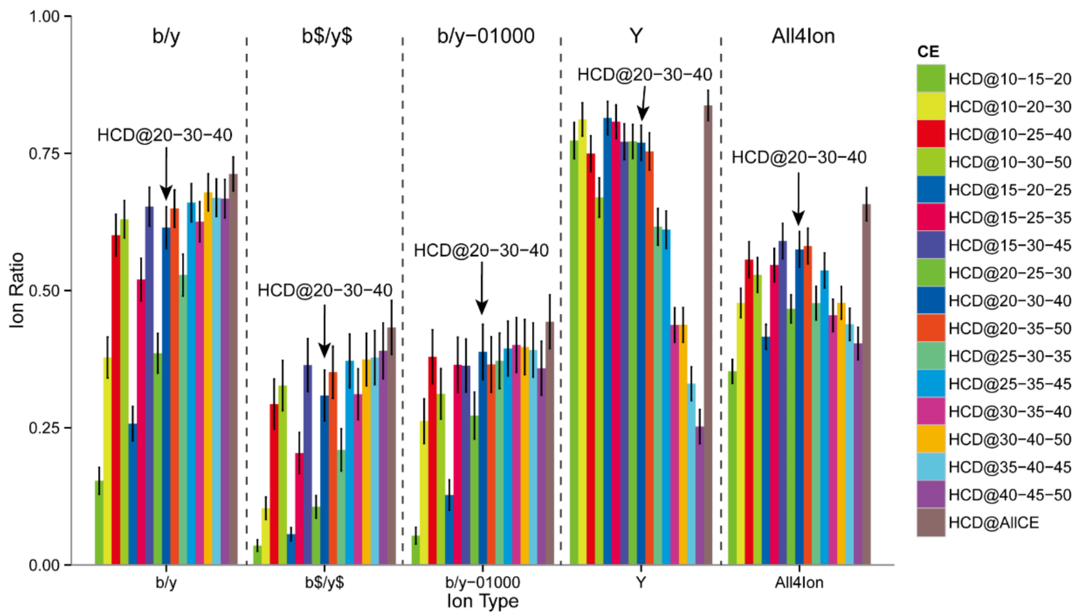

Supplementary Note 1-3. Comparison of 16 possible combinations of SCE in HCD-MS/MS configurations. The normalized matched fragment ion ratio of each ion type is illustrated.

Supplementary Note 2:

Example spectra of glycopeptide fragmentation under different MS/MS conditions

Example spectra of the same glycopeptide (VVLHPJYSQVDIGLIK + Hex × 6 + HexNAc × 5) obtained under 21 different MS/MS collision conditions, including CID/HCD-MS/MS with an energy of 10%, 15%, 20%, 25%, 30%, 35%, 40%, 45%, and 50% and ETD/ETciD/EThcD-MS/MS in their default modes, are shown in the following figures. The glycan composition; peptide sequence, with "J" indicating the N-glycosylation site; and MS/MS collision conditions are shown in the top part of the main box. For peak annotation, green and blue peaks represent the fragment ions of the glycan moiety or the diagnostic glycan ions; red peaks represent the Y ions from glycan fragmentation; and yellow/cyan peaks represent the b/y ions from the peptide backbone fragmentation. For clear illustration, the scale of the relative intensity is automatically adjusted based on the highest peak between 700~2,000 Th. Mass deviations of annotated peaks are shown in the lower box.

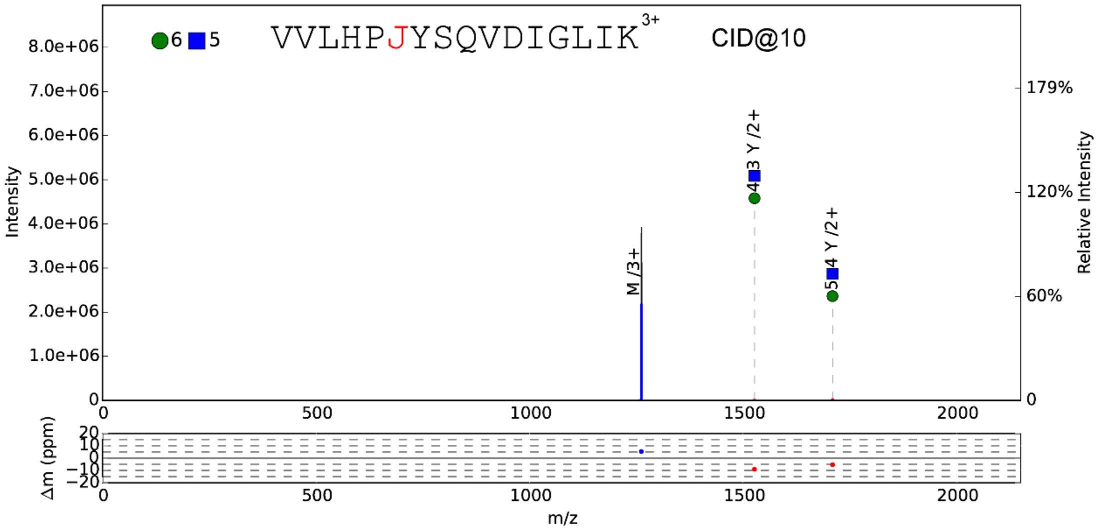

Supplementary Note 2-1. MS/MS collision parameters: CID with an energy of 10%.

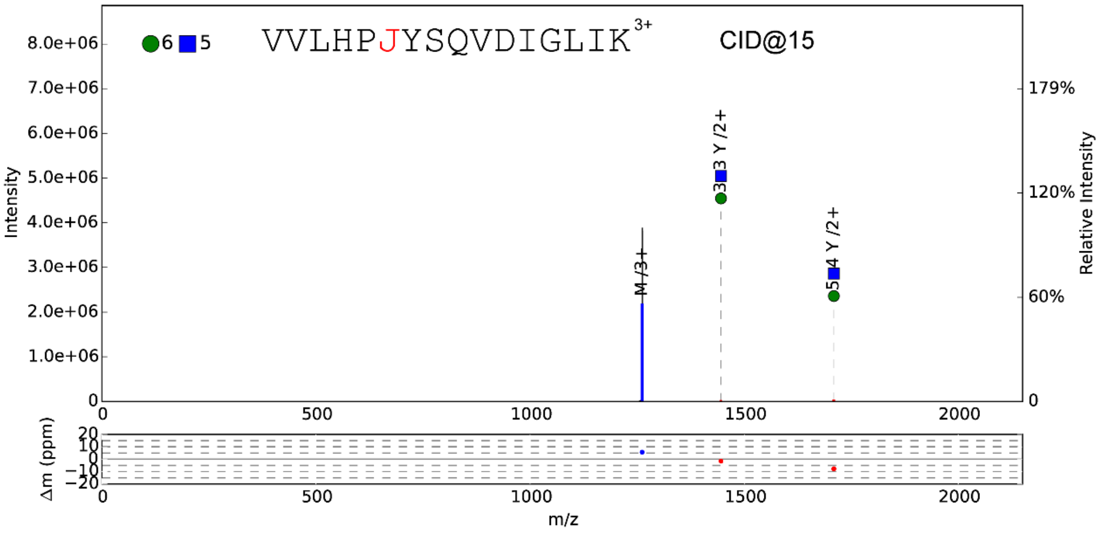

Supplementary Note 2-2. MS/MS collision parameters: CID with an energy of 15%.

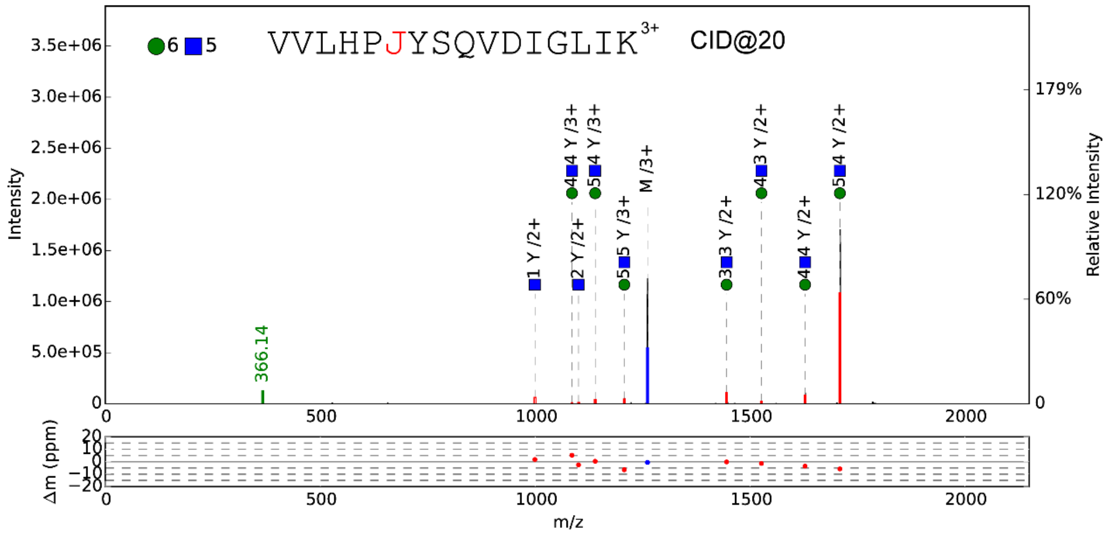

Supplementary Note 2-3. MS/MS collision parameters: CID with an energy of 20%.

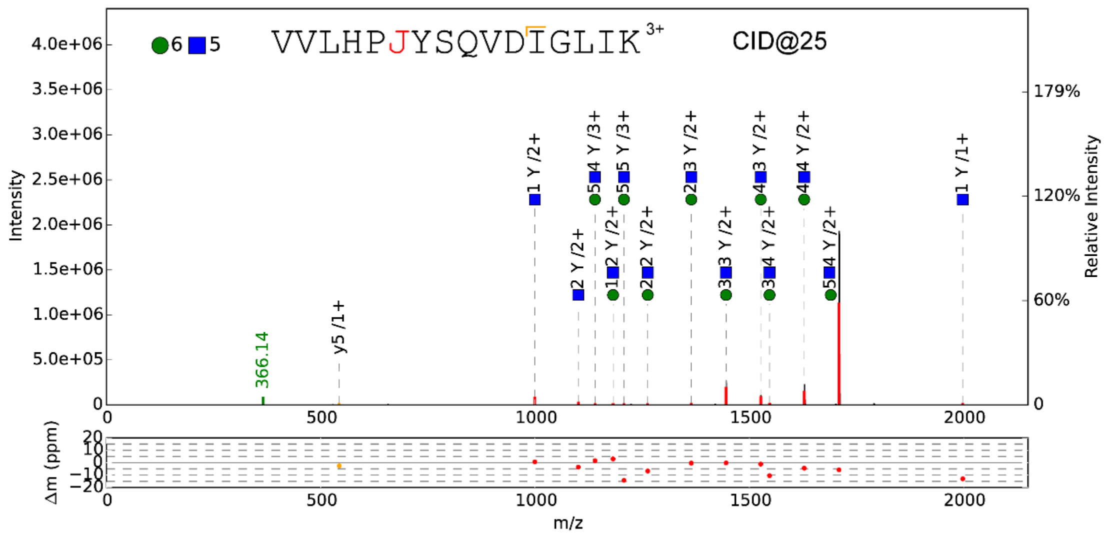

Supplementary Note 2-4. MS/MS collision parameters: CID with an energy of 25%.

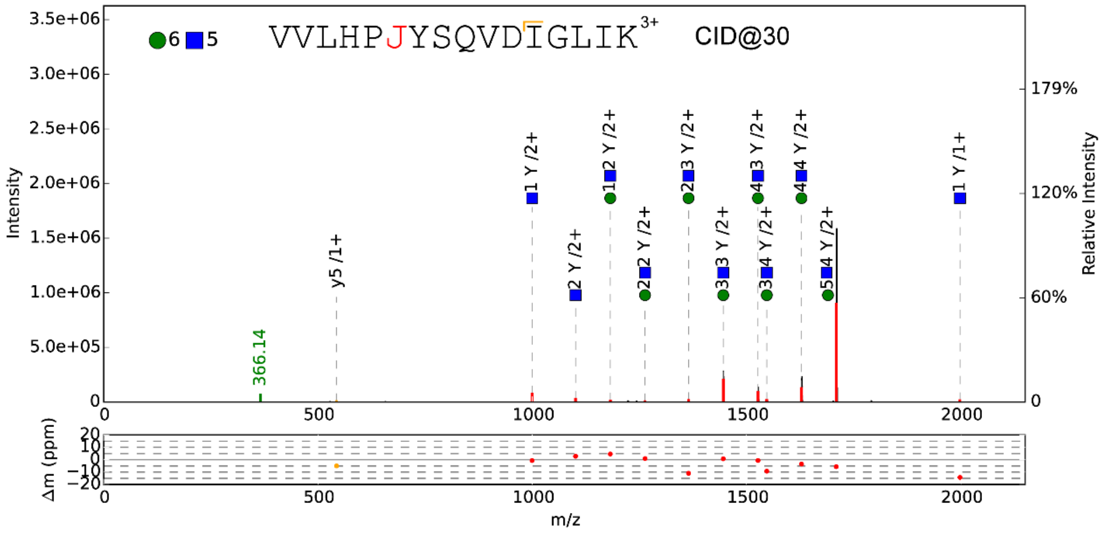

Supplementary Note 2-5. MS/MS collision parameters: CID with an energy of 30%.

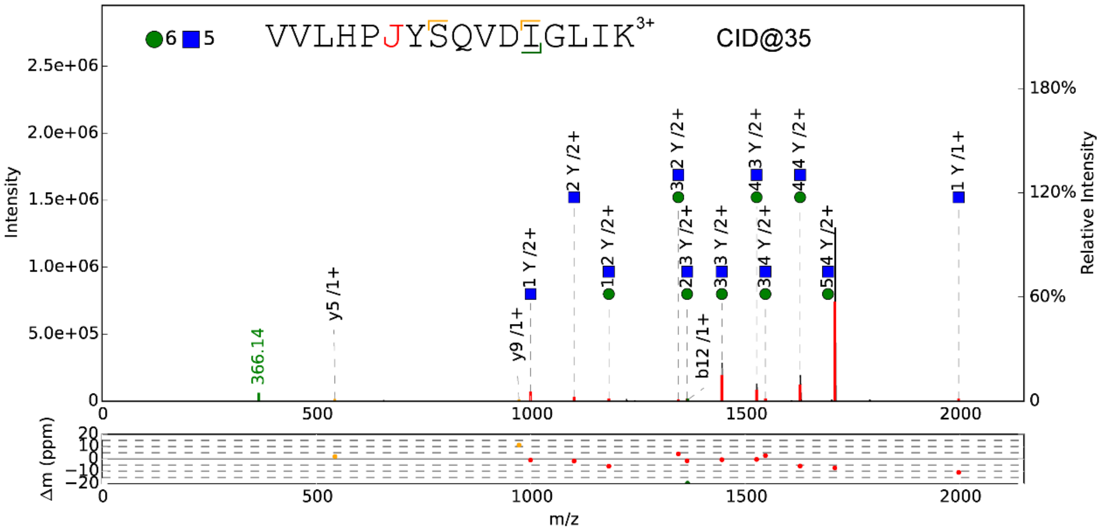

Supplementary Note 2-6. MS/MS collision parameters: CID with an energy of 35%.

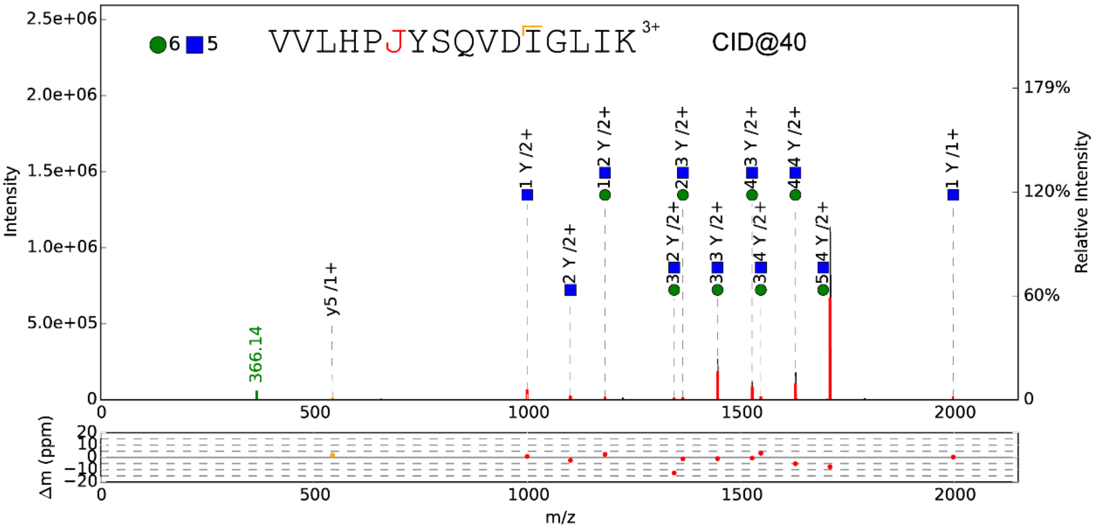

Supplementary Note 2-7. MS/MS collision parameters: CID with an energy of 40%.

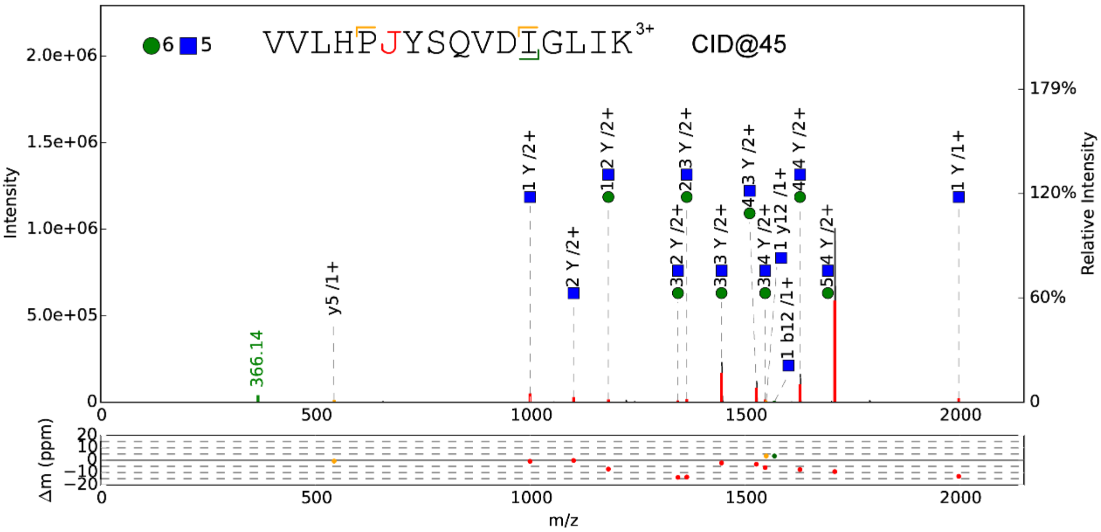

Supplementary Note 2-8. MS/MS collision parameters: CID with an energy of 45%.

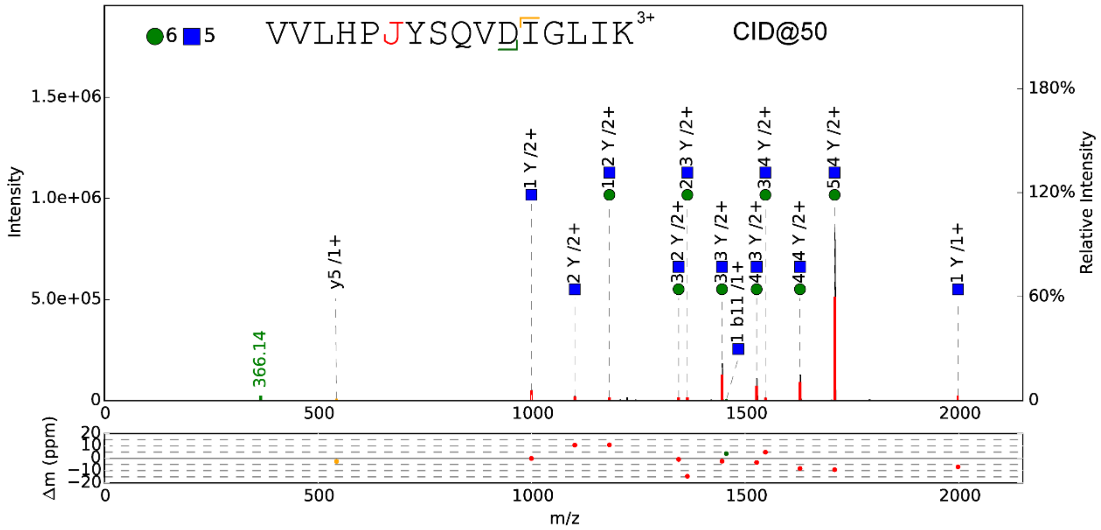

Supplementary Note 2-9. MS/MS collision parameters: CID with an energy of 50%.

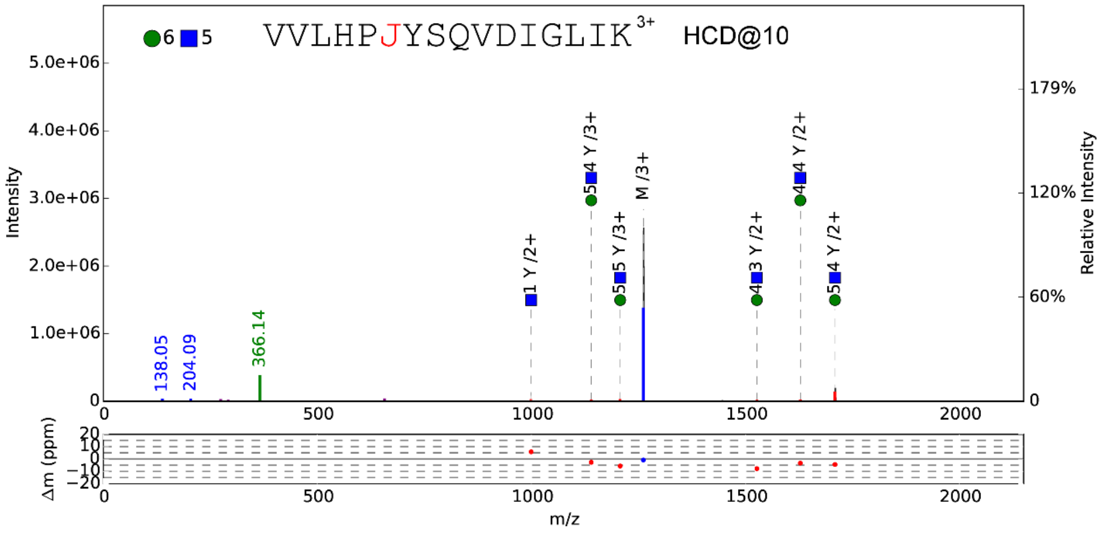

Supplementary Note 2-10. MS/MS collision parameters: HCD with an energy of 10%.

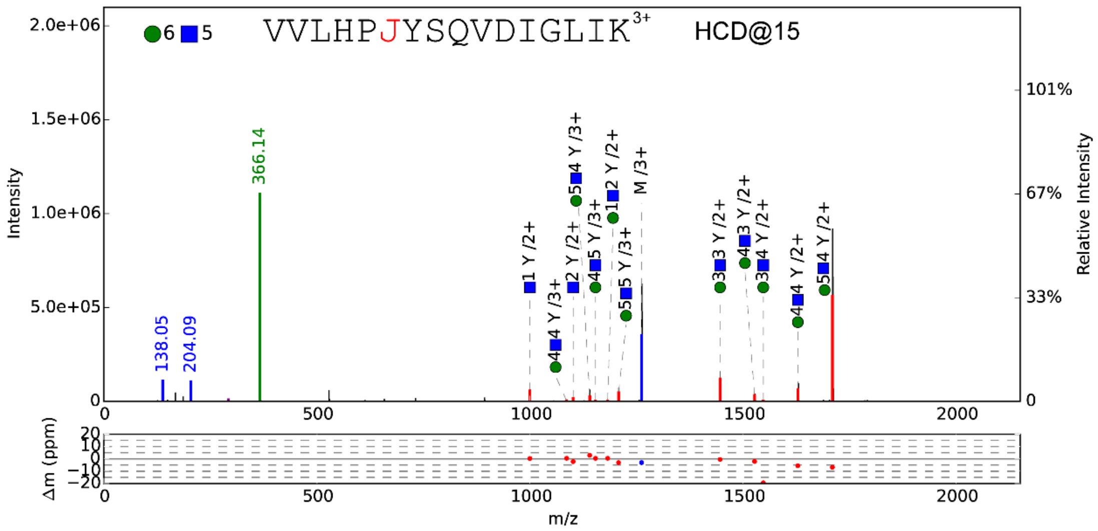

Supplementary Note 2-11. MS/MS collision parameters: HCD with an energy of 15%.

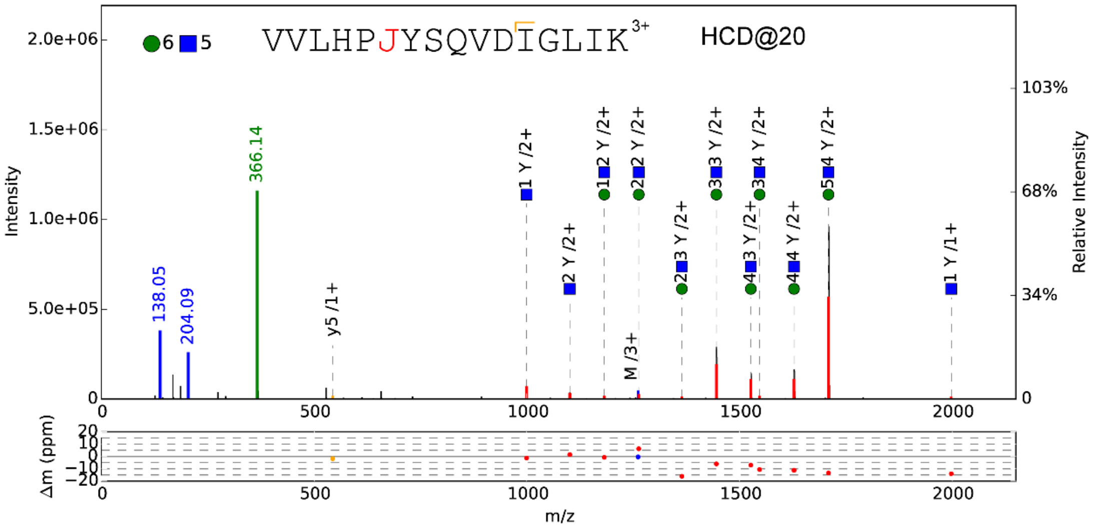

Supplementary Note 2-12. MS/MS collision parameters: HCD with an energy of 20%.

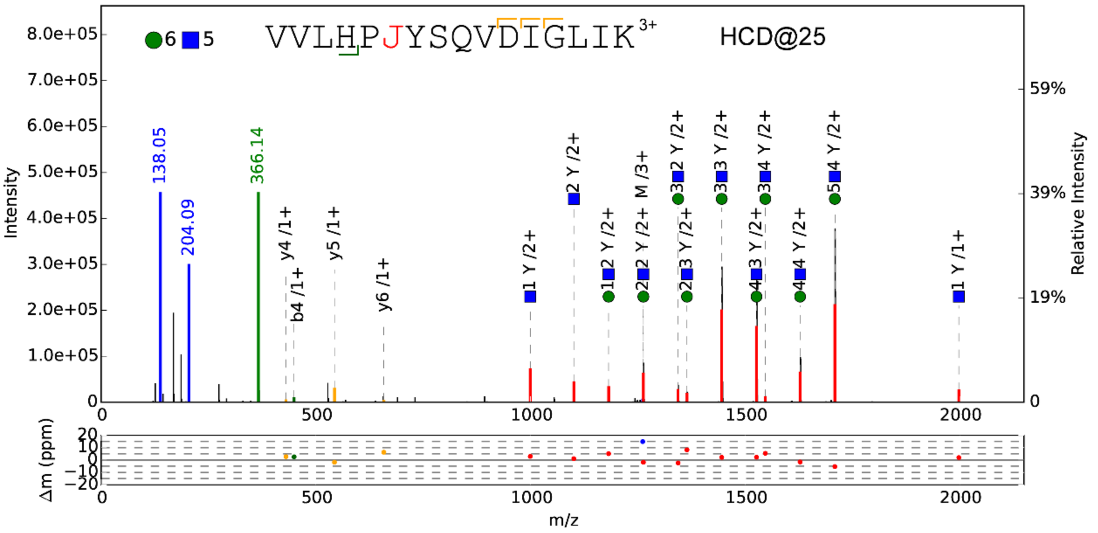

Supplementary Note 2-13. MS/MS collision parameters: HCD with an energy of 25%.

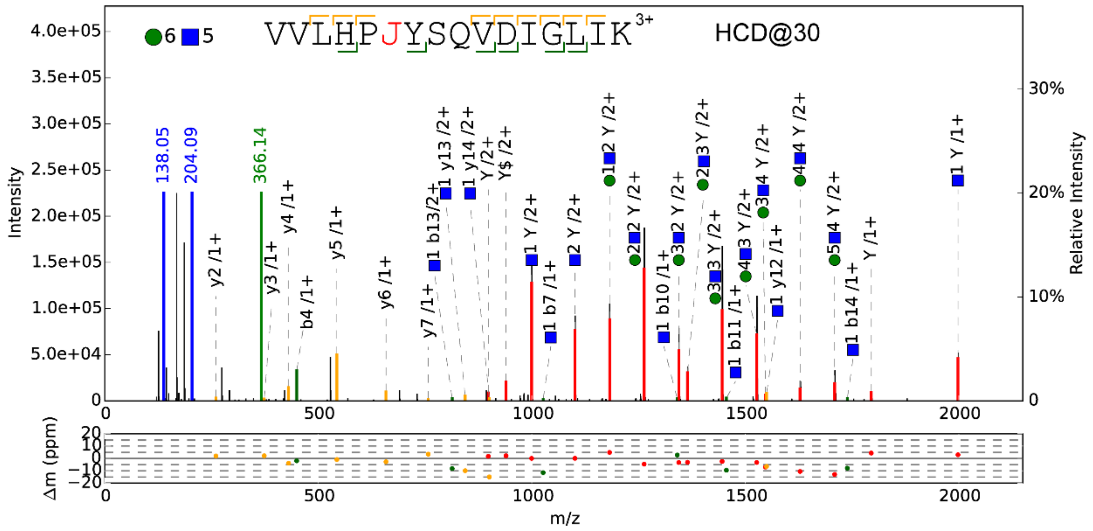

Supplementary Note 2-14. MS/MS collision parameters: HCD with an energy of 30%.

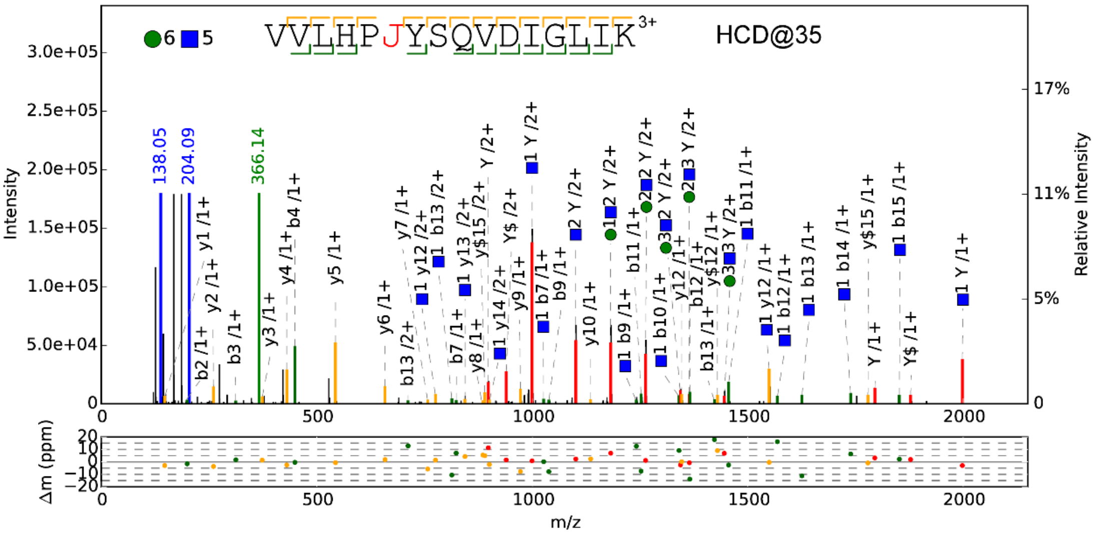

Supplementary Note 2-15. MS/MS collision parameters: HCD with an energy of 35%.

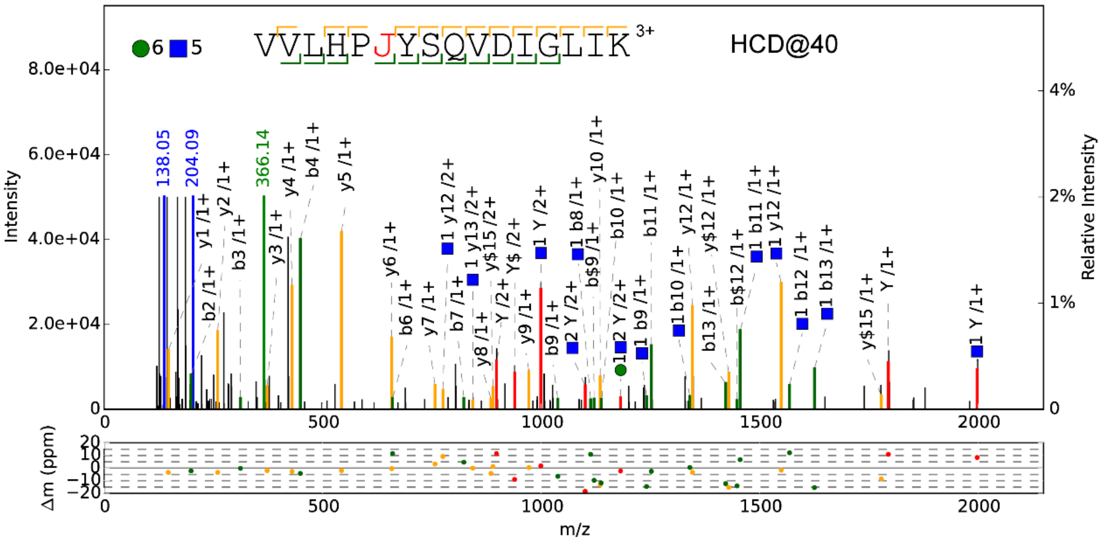

Supplementary Note 2-16. MS/MS collision parameters: HCD with an energy of 40%.

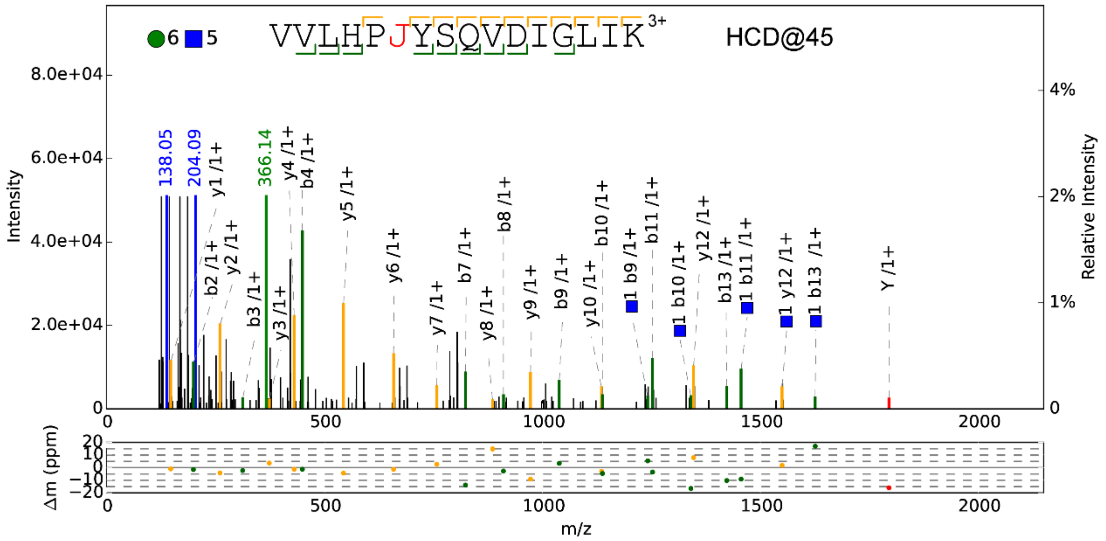

Supplementary Note 2-17. MS/MS collision parameters: HCD with an energy of 45%.

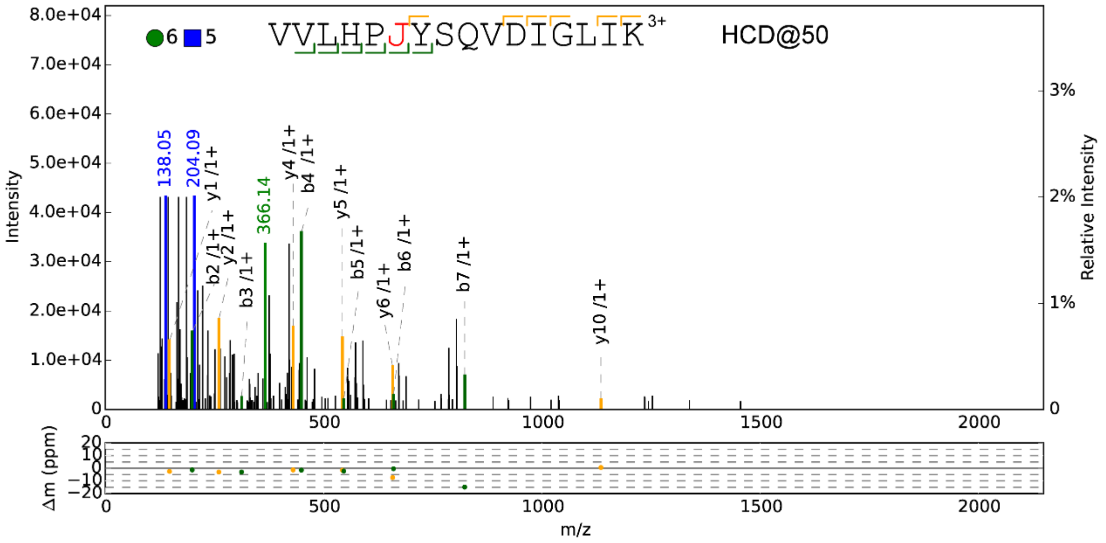

Supplementary Note 2-18. MS/MS collision parameters: HCD with an energy of 50%.

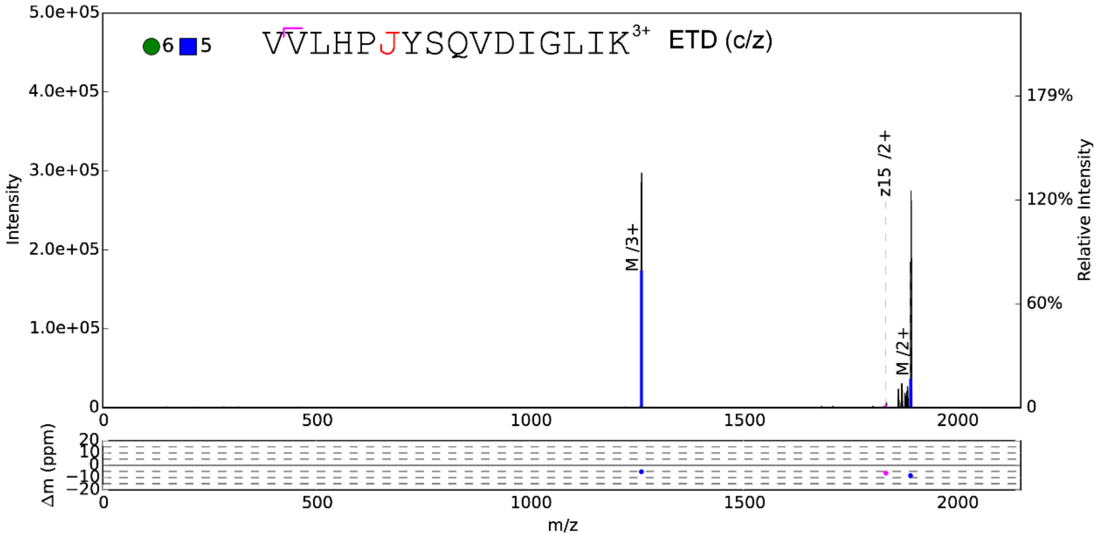

Supplementary Note 2-19. MS/MS collision parameters: ETD; c/z ions are annotated.

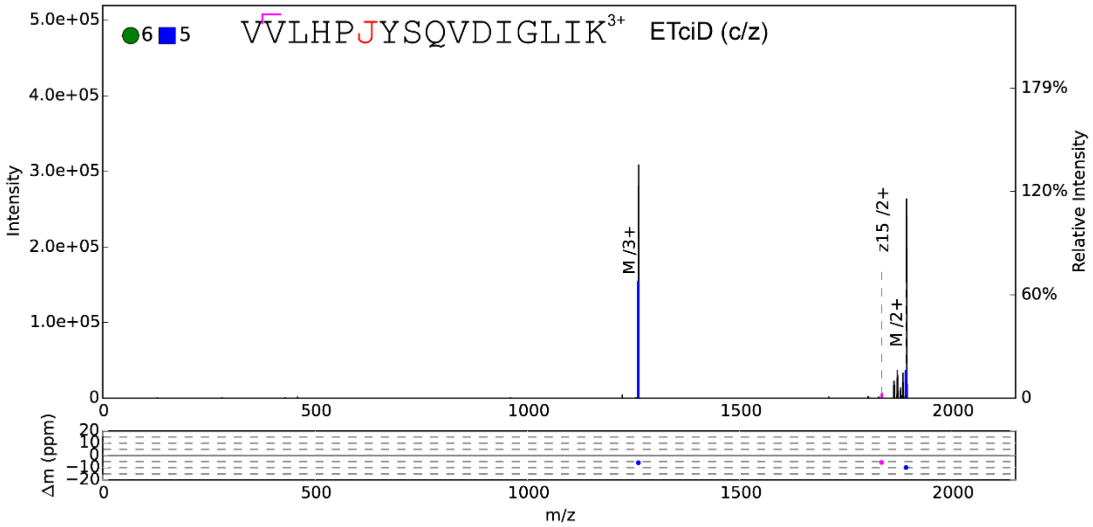

Supplementary Note 2-20. MS/MS collision parameters: ETciD; c/z ions are annotated.

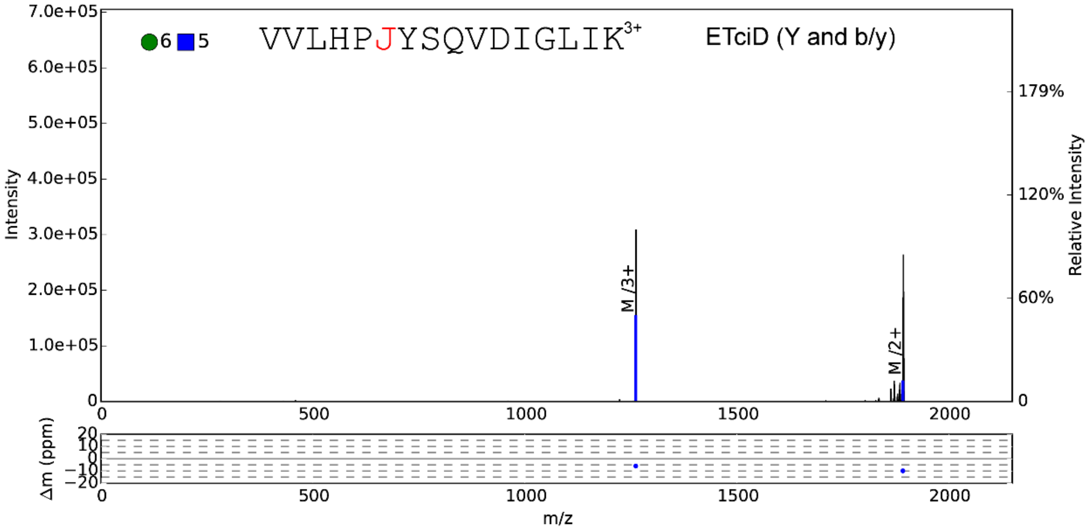

Supplementary Note 2-21. MS/MS collision parameters: ETciD; Y and b/y ions are annotated.

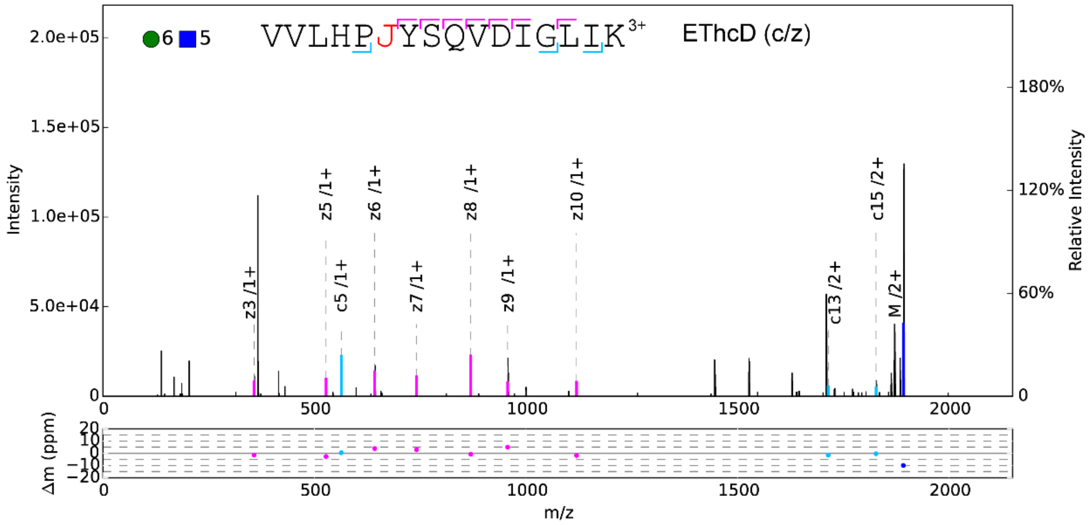

Supplementary Note 2-22. MS/MS collision parameters: EThcD; c/z ions are annotated.

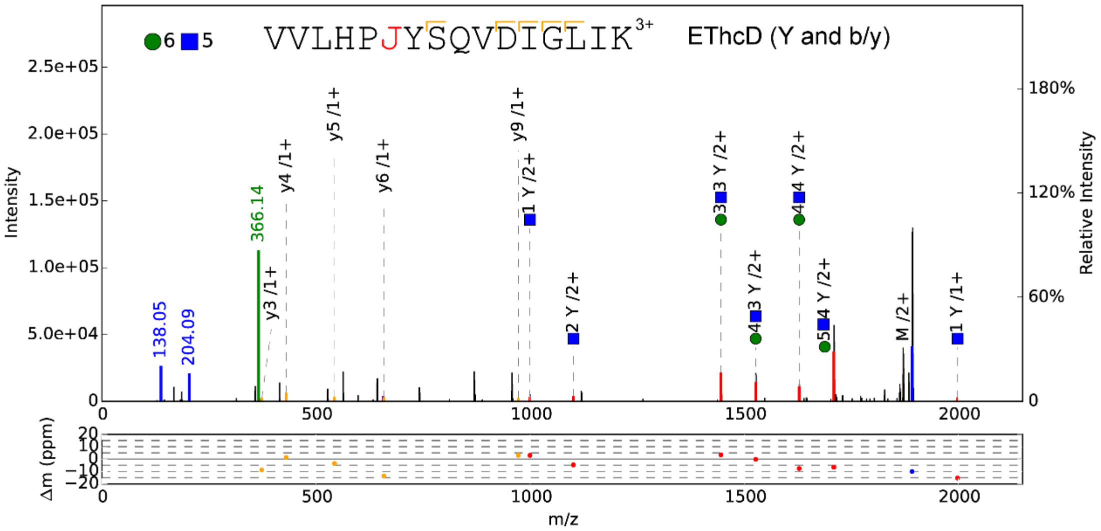

Supplementary Note 2-23. MS/MS collision parameters: EThcD; Y and b/y ions are annotated.

Supplementary Note 3

Comparison of the results from the analysis of a standard glycoprotein mixture with previously reported glycans on the same proteins

We compared the results from the standard glycoprotein mixture analysis in this study with the previously reported glycans on the same proteins. Our results were consistent with those of the previously reported analyses. It is worth mentioning that site-specific glycosylation of the same proteins in different batches of serum/plasma could change partially because glycan synthesis is not a template-driven process[3]. All standard glycoproteins used in our research were from human serum/plasma. The glycan composition in the following table corresponds to the number of Hex / HexNAc / NeuAc / NeuGc / Fuc. For example, 55201 corresponds to a glycan composition of five Hex, five HexNAc, two NeuAc and one Fuc.

Site	Glycan composition	Our results	Ref 3	Ref 4
184	54000			
	54100	Y	Y	Y
	54102	Y		
	54200	Y	Y	Y
	55201		Y	
	63200	Y		
	64101	Y		
	65100	Y		
	65300		Y	
207	54100		Y	

	**54200**	**Y**	**Y**	
**241**	**54000**			**Y**
	**54100**	**Y**		**Y**
	**54101**	**Y**		
	**54102**	**Y**		
	**54200**	**Y**	**Y**	**Y**
	**54201**	**Y**		
	**64100**	**Y**		
	**65000**	**Y**		
	**65100**	**Y**	**Y**	**Y**
	**65200**	**Y**	**Y**	
	**65201**	**Y**	**Y**	
	**65300**	**Y**	**Y**	**Y**
	**65301**	**Y**	**Y**	**Y**
	**76000**	**Y**		
	**76103**	**Y**		
	**76200**	**Y**		
	**76300**	**Y**		
	**86301**	**Y**		

Supplementary Note 3-1. Site-specific glycosylation comparison of protein haptoglobin. “Y” means reported in the form of a glycopeptide.

Site	Glycan composition	Our results	Ref 3
144	44000		Y
	44100		Y
	45000		Y
	54000	Y	Y
	54100		Y
	54101	Y	
	54200		Y
	55000	Y	Y
	55100	Y	Y
	55201	Y	
205	44101	Y	
	45001	Y	Y
	45101	Y	
	54001	Y	Y
	54100	Y	
	54101	Y	Y
	54102	Y	
	54201	Y	Y
	55001	Y	Y
	55002	Y	
	55101	Y	Y
	55102	Y	
	55201	Y	Y
	64101	Y	
	65001	Y	
	65101	Y	

Supplementary Note 3-2. Site-specific glycosylation comparison of protein immunoglobulin A. “Y” means reported in the form of a glycopeptide.

Glycan composition	α2-macroglobulin	Immuno-globulin A	Immuno-globulin G	Immuno-globulin M
34000			3	
34001			13	
35000			1	
35001			4	
43001				2
43100	1			(3)
43101				12
44000			3	
44001			18	3
44101		1	4	2
45000				1
45001		(1)	(5)	(2)
45101		1		5
52000	2			2
53100	1			(3)
53101				(3)
54000		1	2	3
54001		2	10	8
54100	1	2	1	7
54101		6	4	26
54102		(2)		1
54103				(2)
54200				3
54201		5		5
55000		1		1
55001		3	3	2
55002		2		(1)
55100		1		3
55101		4		19
55102		(1)		(2)
55200				(1)
55201		5		3
62000				4
63000	1			1
63001				1

**63100**	**1**			**(6)**
**63101**				**(6)**
**6400**		**(1)**		**(2)**
**64001**				**(2)**
**64100**				**(1)**
**64101**		**(2)**		**(2)**
**64102**				**(2)**
**64200**				**(1)**
**65001**		**2**		
**65100**				**1**
**65101**		**2**		**(4)**
**65102**				**(1)**
**72000**				**2**
**74100**				**(1)**
**82000**				**2**
**92000**				**2**

Supplementary Note 3-3. Protein-specific glycan comparison of α2-macroglobulin and immunoglobulin A, G and M with an N-glycan library for high-abundance glycoproteins in serum, reported in reference 5. The numbers in this table correspond to the numbers of identified glycopeptide spectra matched to the protein-specific glycan. The numbers with brackets mean that the protein-specific glycan is not reported in the library in reference 5, and vice versa. In total, 80.2% (231 / 288) of our identified glycopeptide spectra shown here agree with the library in reference 5.

Supplementary Note 4:

Comparison of pGlyco 2.0 with existing software tools for glycopeptide analysis

**Software tool name**	**Generic search engine using proteome and glycome databases**	**Test of intact glycopeptide data from yeast and mouse used in this paper**
pGlyco 2.0	**Yes**	**Yes**
ArMone	No	N/A, requires deglycopeptide data
Byonic	**Yes**	**Yes, result shown in the paper**
GlycoMasterDB	**Yes**	N/A, requires multiple spectra for each precursor
GlycoFragwork	**Yes**	N/A, requires multiple spectra for each precursor
GRIP	No	N/A, requires deglycopeptide data
GlycoPep Detector	No	N/A, requires multiple spectra for each precursor
GPFinder	No	N/A, not designed for tryptic glycopeptide analysis
GPQuest	No	N/A, requires deglycopeptide data
GPS	**Yes**	Error, out of memory
I-GPA	No	N/A, requires multiple spectra for each precursor *1
MAGIC	No	Error, out of memory *2
Protein-Prospector	No	N/A, requires multiple spectra for each precursor
Sweet-Heart	No	N/A, requires multiple spectra for each precursor

Supplementary Note 4-1. Comparison of pGlyco 2.0 with existing software tools for glycopeptide analysis in terms of the generic search applicability.

N/A: not applicable

Requires multiple spectra for each precursor: paired CID/ETD or HCD/ETD spectra were required

*1: The glycan and protein databases in I-GPA are limited to human in the current version.

*2: MAGIC also has a web-based version; however, the size of the input file is limited to 100 MB.

We used recently published software tools to analyze the yeast and mouse glycoproteome MS/MS data shown in the current paper. Only Byonic successfully performed a generic glycopeptide database search using a complete proteome and glycome database. The testing machine was a common desktop computer with an i7 processor, 8 GB of memory and a Windows 7 system.

**Software tool name**	**Independent glycan FDR**	**Decoy peptide-based FDR**	**Comprehensive glycopeptide FDR**
pGlyco 2.0	**Yes**	**Yes**	**Yes**
ArMone	**Yes**	No	No
Byonic	No	**Yes**	No
GlycoMasterDB	No	**Yes**	No
GlycoFragwork	No	**Yes**	No
GRIP	**Yes**	No	No
GlycoPep Detector	No	**Yes**	No
GPFinder	**Yes**	No	No
GPQuest	No	**Yes**	No
GPS	No	**Yes**	No
I-GPA	No	**Yes**	No
MAGIC	No	**Yes**	No
Protein-Prospector	No	**Yes**	No
Sweet-Heart	No	**Yes**	No

Supplementary Note 4-2. Comparison of pGlyco 2.0 with existing software tools for glycopeptide analysis in terms of the FDR evaluation.

Comprehensive glycopeptide FDR: for a comprehensive glycopeptide FDR analysis, both an independent glycan FDR and a peptide FDR, as well as a method for the integration of the glycan and peptide FDRs, are required.

Software tool name	Method of glycan interpretation	Method of peptide interpretation	Reference
pGlyco 2.0	DB searching	DB searching	-
ArMone	DB searching	De-glycopeptide	[6]
Byonic	Mass matching	DB searching	[7]
GlycoMasterDB	DB searching	DB searching	[8]
GlycoFragwork	De novo	DB searching	[4]
GRIP	DB searching	De-glycopeptide	[9]
GlycoPep Detector	Mass matching	DB searching	[10]
GPFinder	De novo	Mass matching	[11]
GPQuest	Mass matching	De-glycopeptide	[1,12]
GPS	Mass matching	DB searching	[13]
I-GPA	DB searching	DB searching	[14]
MAGIC	De novo	DB searching	[15]
Protein-Prospector	Mass matching	DB searching	[16]
Sweet-Heart	De novo	DB searching	[17,18]

Supplementary Note 4-3. Comparison of pGlyco 2.0 with existing software tools for glycopeptide analysis in terms of the interpretation method.

DB searching: database searching

Mass matching: glycan mass or a few fragments (e.g., Y0, Y1, and Y2) are considered during the spectral analysis

Deglycopeptide: using a deglycopeptide database or spectra

Supplementary Note 5

LC-MS/MS parameter optimization for the large-scale analysis of intact glycopeptides

We tested the effect of several LC-MS/MS parameters on the large-scale analysis of intact glycopeptides in complex samples. Mouse brain, kidney, liver and lung tissues were used as the starting materials for site-specific glycoproteome analysis (Methods). For the analysis of MS/MS data, independent database searches of glycopeptides and regular peptides were carried out using pGlyco and pFind, respectively (Methods).

**Sample**		**Mouse brain**				
**Parameters**	**LC time (hour)**	**2**			**4**	
	**MS/MS accumulation time (ms)**	**125**	**250**	**500**	**250**	**500**
**Glycopeptide identification (pGlyco 2.0)**	**# spectra**	**1,618**	**1,791**	**1,622**	**3,227**	**3,065**
	**# glycopeptide**	**1,153**	**1,262**	**1,112**	**1,876**	**1,756**
	**# glycoprotein**	**283**	**291**	**252**	**378**	**337**
**Peptide identification (pFind)**	**# spectra**	**2,975**	**2,468**	**1,761**	**4,270**	**2,912**
	**# peptide**	**1,444**	**1,129**	**790**	**1,677**	**1,122**
	**# protein**	**785**	**657**	**504**	**914**	**671**

Supplementary Note 5-1. Analysis of MS/MS accumulation time. We first compared the effect of the MS/MS accumulation time in SCE-HCD-MS/MS. As demonstrated in Supplementary Figure 5, many glycopeptide fragments in the MS/MS spectrum have inherently low intensity. A longer MS/MS accumulation time will result in more abundant precursor ions and could potentially increase

the S/N ratio of some low-intensity fragments at the cost of lower overall throughput. In 2- and 4-hour LC runs, an MS/MS accumulation time of 250 ms was the best parameter for glycopeptide analysis. For conventional peptide analysis, a longer MS/MS accumulation time (>125 ms) did not show any benefit.

Sample		Mouse liver				
Parameters	LC time (hour)	6			8	
	MS/MS accumulation time (ms)	250	500		250	500
Glycopeptide identification (pGlyco 2.0)	# spectra	4,666	5,052		5,715	6,263
	# glycopeptide	2,048	2,267		2,129	2,505
	# glycoprotein	367	369		378	401
Peptide identification (pFind)	# spectra	6,353	4,669		8,034	5,627
	# peptide	1,939	1,407		2,090	1,546
	# protein	964	755		1,047	819

Supplementary Note 5-2. Analysis of the MS/MS accumulation time. We extended the LC time to 6 and 8 hours. Under these long gradient conditions, MS/MS accumulation times of 250 and 500 ms resulted in similar glycopeptide identifications. At the same time, the performance of the regular peptide interpretation decreased significantly when the MS/MS accumulation time increased from 250 ms to 500 ms.

Sample		Mouse brain			Mouse liver		
Parameters	LC time (hour)	2	4	6	4	6	8
	MS/MS accumulation time (ms)	250			250		
Glycopeptide identification (pGlyco 2.0)	# spectra	1,791	3,227	4,701	3,622	4,666	5,715
	# glycopeptide	1,262	1,876	2,303	1,876	2,048	2,129
	# glycoprotein	291	378	416	348	367	378
Peptide identification (pFind)	# spectra	2,468	4,270	5,809	4,661	6,353	8,034
	# peptide	1,129	1,677	1,903	1,741	1,939	2,090
	# protein	657	914	1,006	864	964	1,047

Supplementary Note 5-3. Analysis of the total LC time. Next, we tested the effect of the LC time. For complex samples such as mouse tissues, 6 hours is the preferred time. The number of identified unique glycopeptides only increased by 3.9% (2,048 vs 2,129) when the total LC time was increased from 6 hours to 8 hours in the analysis of mouse liver.

Sample		Mouse liver			
Parameters	LC time (hour)	4			
	MS/MS accumulation time (ms)	250			
	Starting material (μg)	50	100	200	400
Glycopeptide identification (pGlyco 2.0)	# spectra	3,719	3,953	3,827	3,964
	# glycopeptide	2,265	2,280	2,099	2,044
	# glycoprotein	400	393	391	377
Peptide identification (pFind)	# spectra	7,762	8,626	8,931	10,225
	# peptide	3,050	3,007	2,911	2,920
	# protein	1,432	1,379	1,338	1,298

Supplementary Note 5-4. Analysis of the starting material. The effect of the starting material amount was also evaluated. LC-MS/MS runs using 50, 100, 200 and 400 μg of sample (before enrichment) were conducted. In our experience, 100 μg of starting material corresponds to approximately 0.5 μg of the glycopeptide mixture injected into the LC-MS/MS instrument. As the starting material amount increased, the number of identified peptide spectra increased, peaking at 100 μg, which suggests that the ionization suppression from regular peptides to glycopeptides was considerable.

Sample		Mouse kidney					
Parameters	LC time (hour)	6					
	MS/MS accumulation time (ms)	250					
	Starting material (μg)	100					
	Replicate	1	2	3	4	5	6
Glycopeptide identification (pGlyco 2.0)	# glycopeptide	2,848	3,276	3,504	3,684	3,882	4,008
	relative ratio	71.1%	81.7%	87.4%	91.9%	96.9%	100.0%
Peptide identification (pFind)	# peptide	1,530	1,758	1,872	1,943	1,999	2,030
	relative ratio	75.4%	86.6%	92.2%	95.7%	98.5%	100.0%

Supplementary Note 5-5. Analysis of the MS reproducibility. Finally, we analyzed the reproducibility of the data-dependent MS acquisition method of glycopeptides and regular peptides by calculating the coverage. The same mouse kidney sample was analyzed 6 times using the same LC-MS/MS parameters. The results of 6 runs were pooled and compared with those of other runs. Note that accumulated result from multiple runs is shown in this table. For example, "replicate 3" means that the accumulated identifications from replicated runs 1, 2 and 3 are shown. To reduce the interference from false positives in the database search (1% FDR), the proteins in the top 99% in terms of abundance were selected for analysis (based on the spectrum count). For glycopeptide identification, the first run identified over 70% of the pooled results and required 5 repetitions to identify over 95% of the pooled result. We also performed the same analysis for regular peptide identification in the

same raw MS files, which required 4 repetitions to identify over 95% of the pooled peptide identifications. The slightly lower reproducibility of the glycopeptide identification relative to the peptide identification was expected, mainly due to the microheterogeneity of glycopeptides and the ionization suppression of peptides.

Supplementary Note 6

Comparison with Byonic on large-scale intact glycopeptide data

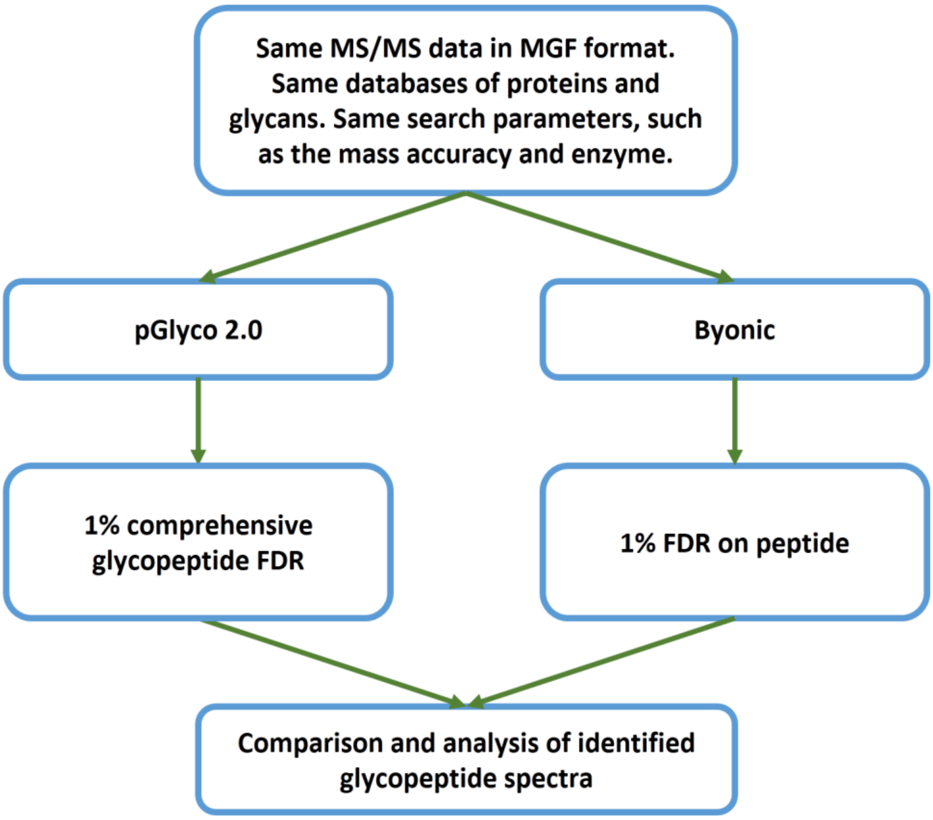

Supplementary Note 6-1. Workflow for the performance comparison between pGlyco 2.0 and Byonic. To ensure an equitable comparison, the same MS/MS data, databases and search parameters (Methods) were used. The MS/MS data consisted of the same MGF file containing data from a large-scale intact N-glycopeptide analysis of mouse tissues. For the FDR analysis of peptides in the Byonic results, peptides that matched to the target mouse protein database were considered "true matches", and peptides that matched to the yeast protein database were considered "false matches". The FDR was calculated

as the number of "false matches" divided by the number of all matched spectra.

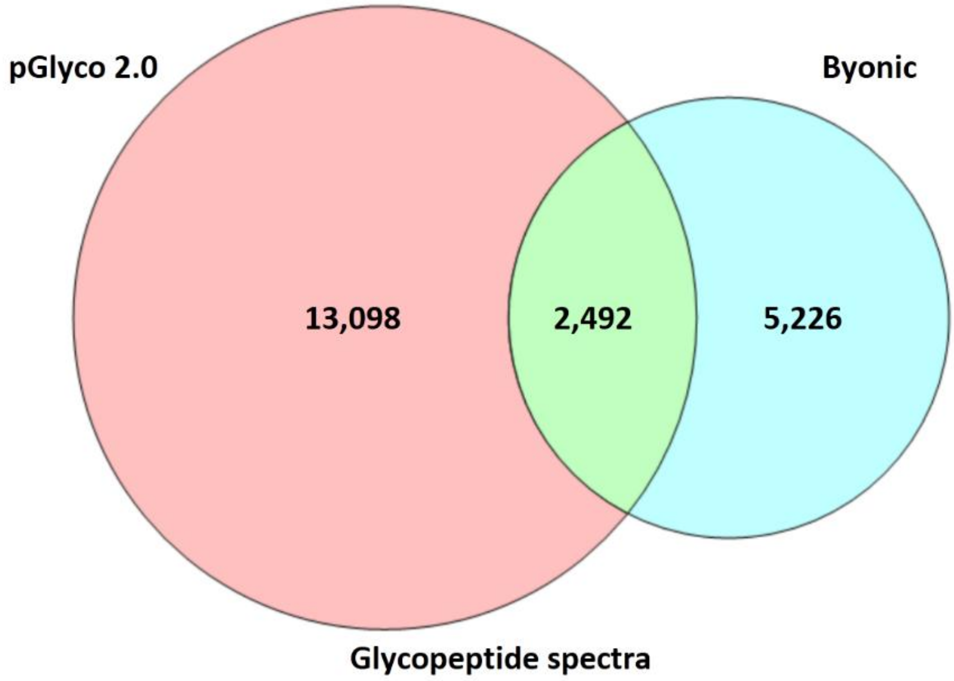

Supplementary Note 6-2. Results of the performance comparison between pGlyco 2.0 and Byonic. The glycopeptides identified from the spectral peptides are shown in the Venn diagram.

Interestingly, the overlap of the glycopeptide spectra was quite low: pGlyco 2.0 only covered 32.3% of the spectra reported by Byonic (2,492 / 7,718). We analyzed the 5,226 Byonic-only glycopeptide identifications and found several different scenarios. Example spectra are shown in the following section (Supplementary Note 3-3 to Supplementary Note 3-6). The design of the annotation in each spectrum is the same as that in Supplementary Figure 1.

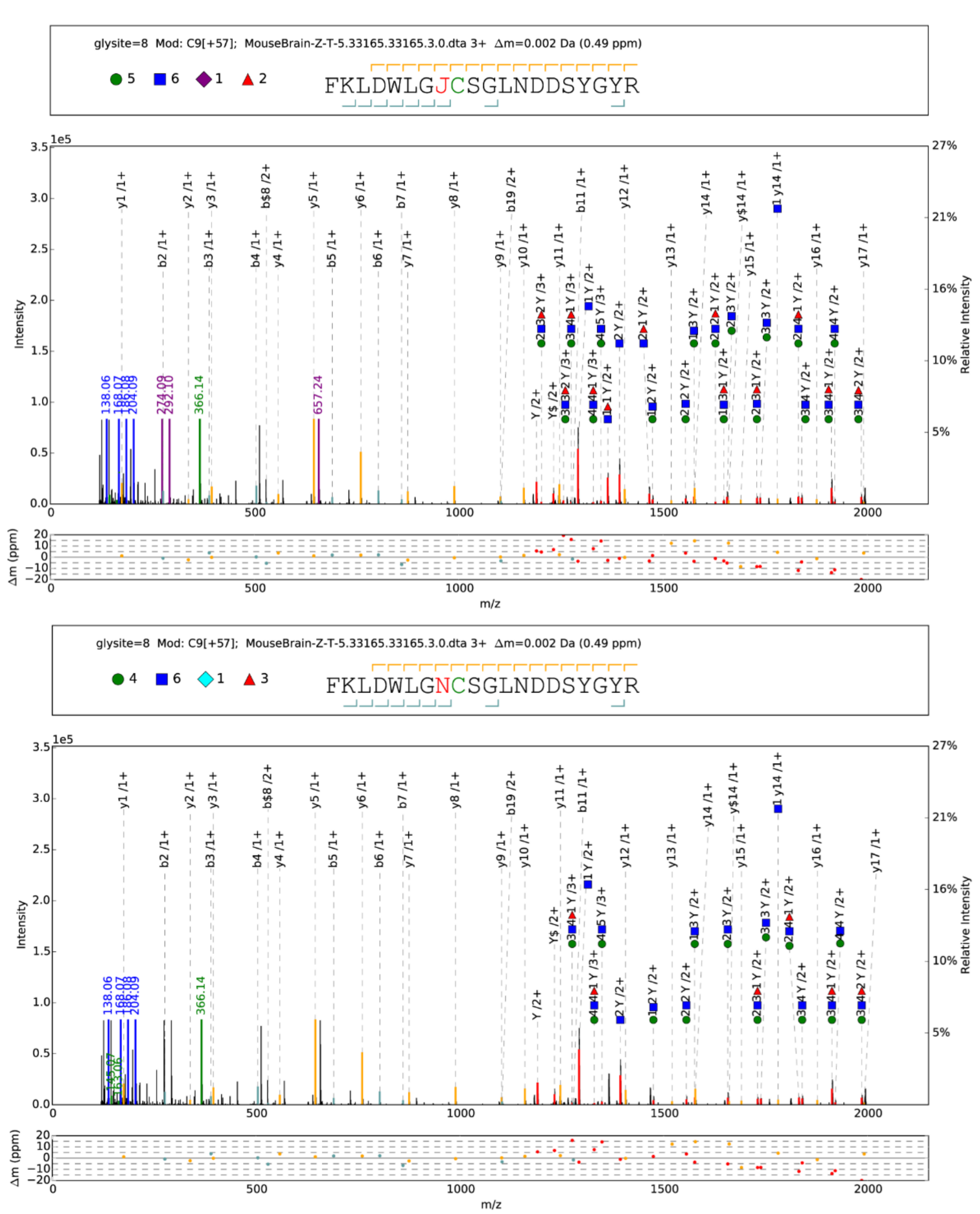

Supplementary Note 6-3. Examples of different glycopeptide identifications made by pGlyco 2.0 and Byonic. (a) Glycopeptide identification reported by pGlyco 2.0. (b) Glycopeptide identification reported by Byonic for the same spectrum.

This glycopeptide spectrum only contains diagnostic ions of NeuAc (274.09,

292.10 and 657.24), but no diagnostic ions of NeuGc were found. Therefore, the glycopeptide identification reported by Byonic, which includes one NeuGc, was likely a false positive. In addition, there were only trace amounts of NeuGc in the mouse brain. Out of 5,226 Byonic-only glycopeptide identifications, 1,090 were reported as NeuGc-containing glycopeptides.

a

b

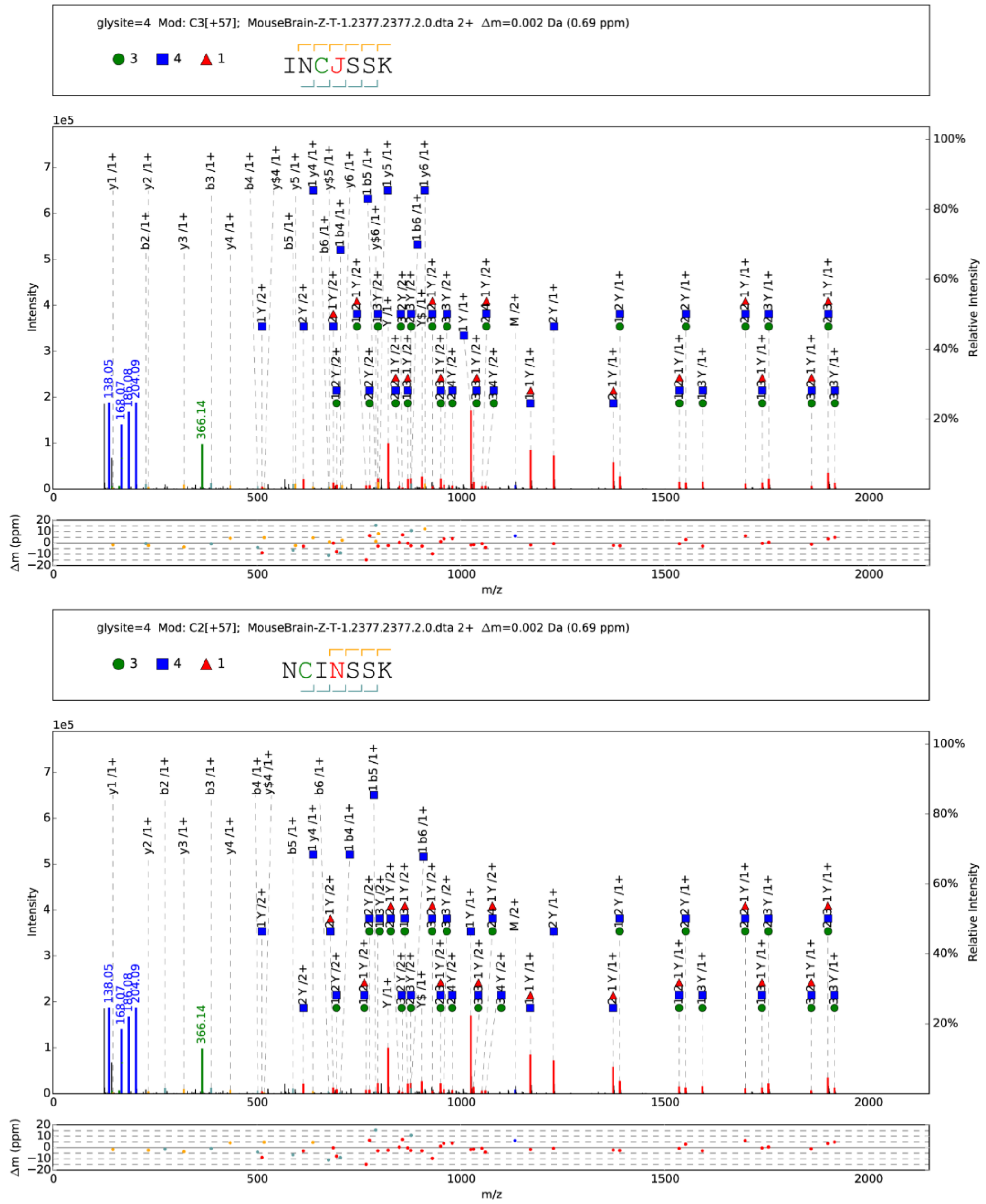

Supplementary Note 6-4. Example of different glycopeptide identifications made by pGlyco 2.0 and Byonic. (a) Glycopeptide identification reported by pGlyco 2.0. (b) Glycopeptide identification reported by Byonic for the same spectrum. In this case, pGlyco 2.0 and Byonic reported glycopeptides with the same glycan and very similar peptide sequences (INCNSSK and NCINSSK by pGlyco 2.0 and Byonic, respectively).

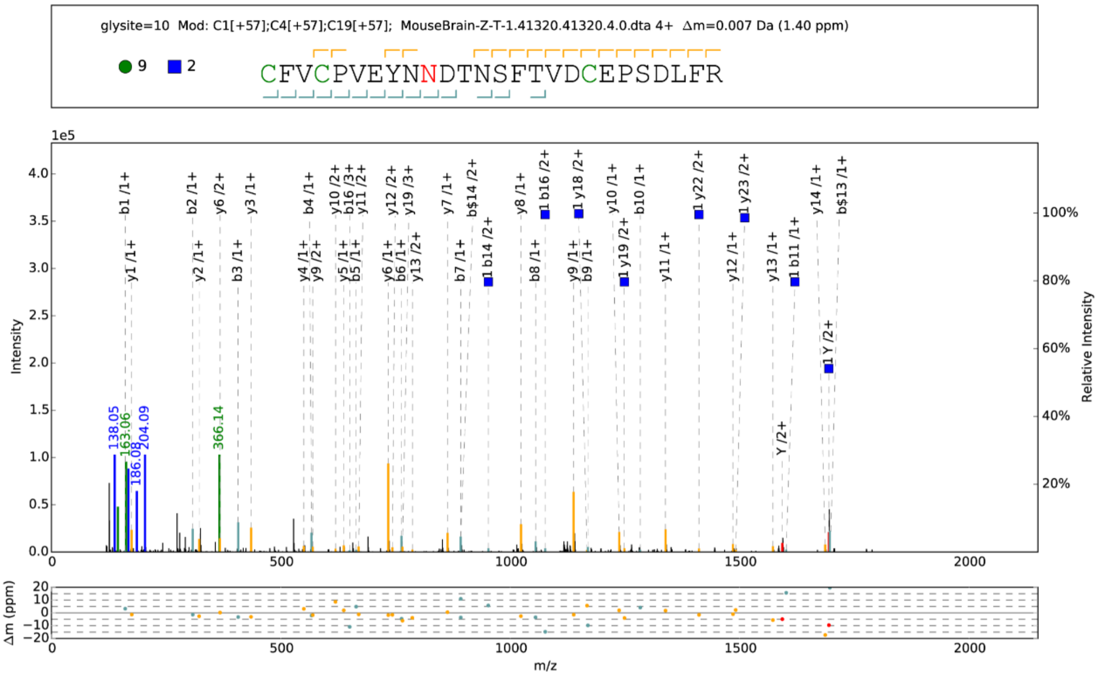

Supplementary Note 6-5. Example of different glycopeptide identifications made by pGlyco 2.0 and Byonic. This glycopeptide identification was reported by Byonic. pGlyco 2.0 found the same glycopeptide candidate in the database search. However, there are very few glycan fragments in the spectrum, and pGlyco 2.0 filtered out this glycopeptide identification in the FDR analysis.

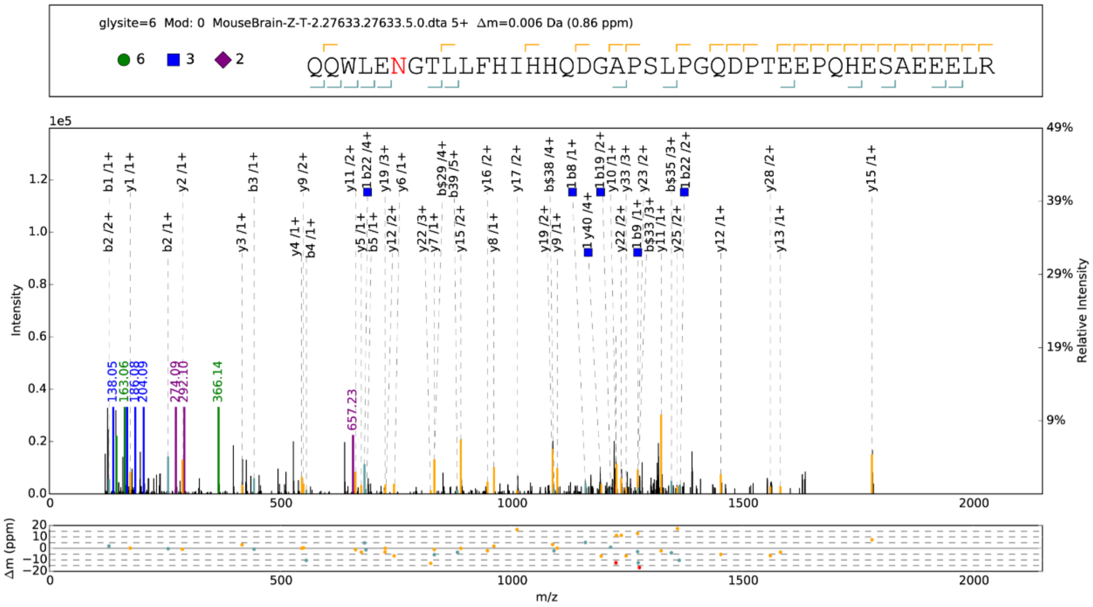

Supplementary Note 6-6. Example of different glycopeptide identifications made by pGlyco 2.0 and Byonic. This glycopeptide identification was reported by Byonic. pGlyco 2.0 did not find the same glycopeptide candidate in the database search possibly because of the absence of glycan fragments.

For the glycopeptides shown in Supplementary Note 6-5 and 6-6, the energies used in our SCE-HCD-MS/MS method may not have been optimal, as discussed in Supplementary Figure 2.

Supplementary References

1. Sun, S. et al. Comprehensive analysis of protein glycosylation by solid-phase extraction of N-linked glycans and glycosite-containing peptides. Nature biotechnology 34, 84-88 (2016).

2. Zielinska, D.F., Gnad, F., Wisniewski, J.R. & Mann, M. Precision mapping of an in vivo N-glycoproteome reveals rigid topological and sequence constraints. Cell 141, 897-907 (2010).

3. Hong, Q.T. et al. A Method for Comprehensive Glycosite-Mapping and Direct Quantitation of Serum Glycoproteins. Journal of proteome research 14, 5179-5192 (2015).

4. Mayampurath, A. et al. Computational framework for identification of intact glycopeptides in complex samples. Analytical chemistry 86, 453-463 (2014).

5. Aldredge, D., An, H.J., Tang, N., Waddell, K. & Lebrilla, C.B. Annotation of a Serum N-Glycan Library for Rapid Identification of Structures. Journal of proteome research 11, 1958-1968 (2012).

6. Cheng, K. et al. Large-scale characterization of intact N-glycopeptides using

an automated glycoproteomic method. Journal of proteomics 110, 145-154 (2014).

7. Medzihradszky, K.F., Maynard, J., Kaasik, K. & Bern, M. Intact N- and O-linked Glycopeptide Identification from HCD Data Using Byonic. Molecular & Cellular Proteomics 13, S36-S36 (2014).

8. He, L., Xin, L., Shan, B., Lajoie, G.A. & Ma, B. GlycoMaster DB: software to assist the automated identification of N-linked glycopeptides by tandem mass spectrometry. Journal of proteome research 13, 3881-3895 (2014).

9. Liu, M. et al. Efficient and accurate glycopeptide identification pipeline for high-throughput site-specific N-glycosylation analysis. Journal of proteome research 13, 3121-3129 (2014).

10. Zhu, Z., Hua, D., Clark, D.F., Go, E.P. & Desaire, H. GlycoPep Detector: a tool for assigning mass spectrometry data of N-linked glycopeptides on the basis of their electron transfer dissociation spectra. Analytical chemistry 85, 5023-5032 (2013).

11. Strum, J.S. et al. Automated Assignments of N- and O-Site Specific Glycosylation with Extensive Glycan Heterogeneity of Glycoprotein Mixtures.

Analytical chemistry 85, 5666-5675 (2013).

12. Eshghi, S.T., Shah, P., Yang, W.M., Li, X.D. & Zhang, H. GPQuest: A Spectral Library Matching Algorithm for Site-Specific Assignment of Tandem Mass Spectra to Intact N-glycopeptides. Analytical chemistry 87, 5181-5188 (2015).

13. Chandler, K.B., Pompach, P., Goldman, R. & Edwards, N. Exploring Site-Specific N-Glycosylation Microheterogeneity of Haptoglobin Using Glycopeptide CID Tandem Mass Spectra and Glycan Database Search. Journal of proteome research 12, 3652-3666 (2013).

14. Park, G.W. et al. Integrated GlycoProteome Analyzer (I-GPA) for Automated Identification and Quantitation of Site-Specific N-Glycosylation. Sci Rep-Uk 6 (2016).

15. Lynn, K.S. et al. MAGIC: An Automated N-Linked Glycoprotein Identification Tool Using a Y1-Ion Pattern Matching Algorithm and in Silk MS2 Approach. Analytical chemistry 87, 2466-2473 (2015).

16. Medzihradszky, K.F., Kaasik, K. & Chalkley, R.J. Tissue-Specific Glycosylation at the Glycopeptide Level. Molecular & Cellular Proteomics 14, 2103-2110 (2015).

17. Wu, S.W., Liang, S.Y., Pu, T.H., Chang, F.Y. & Khoo, K.H. Sweet-Heart - An integrated suite of enabling computational tools for automated MS2/MS3 sequencing and identification of glycopeptides. Journal of proteomics 84, 1-16 (2013).

18. Wu, S.W., Pu, T.H., Viner, R. & Khoo, K.H. Novel LC-MS(2) product dependent parallel data acquisition function and data analysis workflow for sequencing and identification of intact glycopeptides. Analytical chemistry 86, 5478-5486 (2014).
